# Insights into the complexity of SARS-CoV-2 M^pro^ inhibition: Ebselen and its derivatives impair dimerisation of the enzyme

**DOI:** 10.1080/14756366.2025.2604232

**Published:** 2026-01-02

**Authors:** Simone Fabbian, Silvia Fabi, Laurin Schwarz, Giovanni Preto, Chiara Schiavinato, Cristiano Salata, Letizia Crocetti, Roberto Battistutta, Barbara Gatto, Alice Sosic

**Affiliations:** ^a^ Department of Pharmaceutical and Pharmacological Sciences, University of Padova, Padova, Italy; ^b^ Department of Molecular Medicine, University of Padova, Padova, Italy; ^c^ NEUROFARBA, Pharmaceutical and Nutraceutical Section, University of Florence, Sesto Fiorentino, Italy; ^d^ Department of Chemical Sciences, University of Padova, Padova, Italy

**Keywords:** Ebselen, organoselenium derivatives, SARS-CoV-2 Mpro, Mpro dimerization, anti-coronavirus agents

## Abstract

The SARS-CoV-2 Main Protease (M^pro^), a key enzyme for viral replication, represents a highly attractive target for the development of broad-spectrum anti-coronavirus therapeutics. The organoselenium drug Ebselen has shown potent *in vitro* inhibition of M^pro^ as well as antiviral activity, granting clinical interest as a COVID-19 treatment option. Here we show that Ebselen and selected derivatives with human neutrophil elastase (HNE) inhibition and anti-radical activity are able to bind covalently to the viral enzyme with multiple stoichiometry, exhibiting inhibitory activity towards SARS-CoV-2 M^pro^ with potencies in the nanomolar range. Employing a mass spectrometry-based approach, we show that, upon binding to the target, Ebselen and its derivatives induce a dose-dependent shift in the dimer-monomer equilibrium, favouring the inactive monomeric state of the viral protease and possibly contributing to the observed *in vitro* inhibition.

## Graphical abstract

**Figure:**
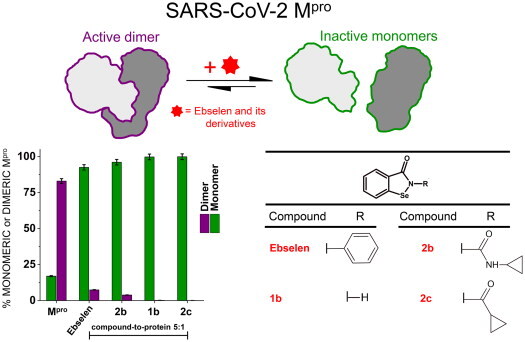

## Introduction

The profound global impact of the COVID-19 pandemic, caused by the severe acute respiratory syndrome coronavirus 2 (SARS-CoV-2), has highlighted the need for sustained research to prevent and be prepared for further severe health emergencies. Among the molecular targets involved in the viral replication cycle that have been identified and validated for effective anti-coronavirus therapies, the main protease (M^pro^) has emerged as an attractive and promising target due to its essential function, the absence of closely related homologs in humans and the high degree of conservation across coronaviruses^1–3^. SARS-CoV-2 M^pro^, also known as 3-chymotrypsin-like protease (3CL^pro^) or nsp5, is a cysteine protease that functions as a homodimer, with each protomer comprising three distinct domains. Catalysis is mediated by a conserved catalytic dyad comprising Histidine 41 (H41) and Cysteine 145 (C145), located at the interface between domains I (residues 10–99) and II (residues 100–182)^4^. Domain III (residues 198–303), which contains five α-helices arranged into a globular cluster^4^, is instead required for the dimerisation of the viral protein, necessary for a fully active enzyme^4–6^. M^pro^ plays a pivotal role in the viral replication by cleaving the virus-encoded polyproteins pp1a and pp1ab at specific and conserved sites to release 13 of the 16 non-structural proteins (nsps), which are critical for viral genome transcription and replication within the host^3^.

**Figure 1.**
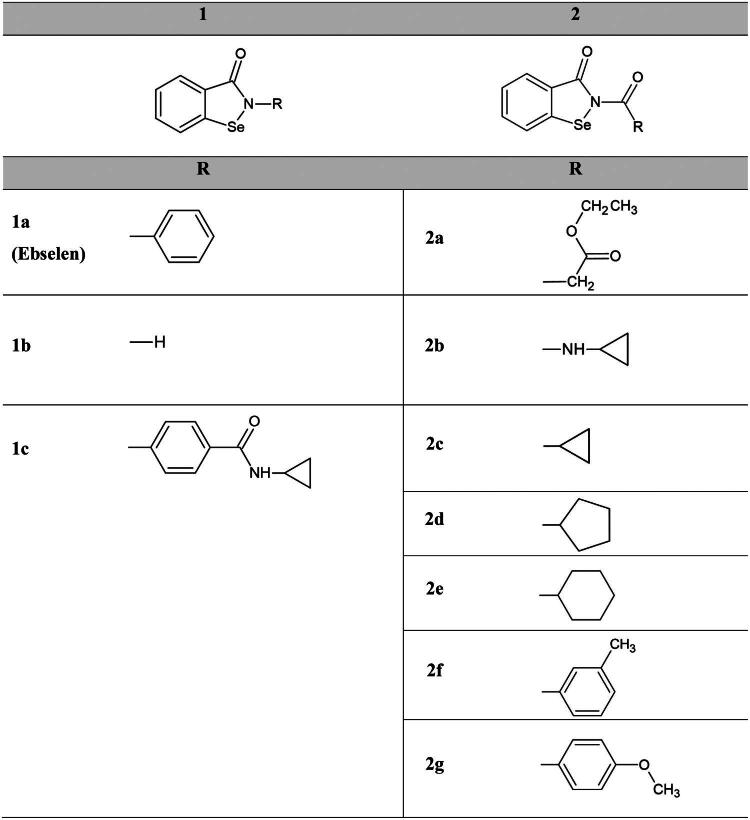
Chemical structure of Ebselen and its derivatives analysed in this study. Series 1 details Ebselen and related structures, while series 2 identifies benzoselenazolones N-acyl-derivatives.

Multiple approaches have been employed to identify SARS-CoV-2 M^pro^ inhibitors. Several drug candidates have been proposed, among them peptidomimetics^7^, which share mechanistic similarities with the approved drug Nirmatrelvir^8^, and several structurally distinct small molecules identified through different drug discovery efforts^4^^,^^9–13^. Ebselen (Figure 1), an organoselenium compound with antioxidant, anti-inflammatory, antiviral and cytoprotective properties^14^^,^^15^, is a versatile molecule currently in clinical trials for hearing-related disorders^16^. Identified as M^pro^ inhibitor, Ebselen has been proposed for COVID 19 patients^16^, driving the interest for its development as anti-coronavirus drug: several compounds sharing ebselen scaffold have indeed been synthesised and their activity tested by different groups^17–20^. Among them, two series of recent derivatives showed dual human neutrophil elastase (HNE) inhibition and anti-radical activity^21^. Since excessive HNE activity is responsible for disease exacerbation and severe respiratory complications in COVID patients^22^, it was proposed to test these new ebselen derivatives for M^pro^ inhibition^21^. On this basis, we selected from the library the compounds shown in Figure 1 and investigated their potential inhibition against SARS-CoV-2 M^pro^. We show here that all tested compounds exhibit potent enzyme inhibition *in vitro*, binding covalently to reactive sites in the enzyme with different binding stoichiometry already at limiting concentrations. More interestingly, we demonstrate that binding by Ebselen and tested derivatives shifts the dimer-monomer equilibrium of the enzyme towards the inactive monomeric form, hinting to an additional inhibition mode possibly involving recognition of multiple sites of the protein.

## Materials and methods

### Compounds and chemical reagents

Ebselen and its analogues (Figure 1) were synthesised in-house. The structures were confirmed on the basis of analytical and spectral data. All final compounds were characterised by ^1^H-NMR,^13^C-NMR,^77^Se-NMR and ITMS (ESI) to confirm structures and purity, with most showing a purity > 95%. In addition, the melting points of the synthesised Ebselen and its analogues were measured. Briefly, starting from benzoic acid diselenide, the acyl chloride was obtained by treating with an excess of thionyl chloride in toluene under reflux conditions. The latter was treated with the appropriate aniline in anhydrous dichloromethane and triethylamine to obtain compounds **1a** (Ebselen) and **1c**. On the other hand, the treatment of the acyl chloride with NH_3_ gas in tetrahydrofuran led to the benzo[d][1,2]selenazol-3(2H)-one **1b** which was acylated using the appropriate acyl chloride in dichloromethane and triethylamine to obtain the final compounds **2a** and **2c-g**. To obtain ureido compound **2b**, we treated intermediate **1b** with phenyl chloroformate and DIPEA in anhydrous acetonitrile, yielding the corresponding carbamate derivative, which was reacted with cyclopropylamine and DIPEA in the same solvent under reflux and nitrogen flow to obtain the desired compound^21^. Stock solutions of all compounds were prepared at a final concentration of 10 mM in anhydrous DMSO and stored at − 20 °C before using. The water solubility of Ebselen and its analogues was estimated using predicted Log P (SwissADME: http://www.swissadme.ch/index.php) and Log D values at pH 7.4 (ChemAxon: https://plugins.calculators.cxn.io/logd/).

All salts and solvents used for sample preparation were purchased from Sigma-Aldrich (Merck, Darmstadt, Germany).

### Recombinant expression and purification of wild type and double mutant SARS-CoV-2 M^pro^

Synthetic cDNA encoding for wild-type and double mutant SARS-CoV-2 M^pro^ has been synthesised and E. coli codon optimised by Thermofisher Scientific. The sequence encoding wild-type SARS-CoV-2 M^pro^ (wt-M^pro^ corresponding to residues S3264-Q3569 of the SARS-CoV-2 replicase polyprotein 1ab, UniProtKB-P0DTD1, here renumbered S1-Q306) was initially cloned into the Champion^TM^ pET SUMO expression vector (Thermofisher Scientific) by restriction-free cloning. The plasmid encoding the catalytically inactive double mutant (dm-M^pro^), bearing the H41A and C145A mutations within the catalytic dyad, was subsequently generated by single site-directed mutagenesis. The purified plasmids were then transformed into competent *E. coli* BL21 (DE3) cells (Thermofisher Scientific). Following overnight expression at 20 °C, the cleared lysate was resuspended in 98% buffer A (50 mM sodium phosphate, 300 mM NaCl, pH 7.8) with 2% buffer B (50 mM sodium phosphate, 300 mM NaCl, 500 mM imidazole, pH 7.8) and then loaded onto a 1 ml affinity Ni^2+^-HisTrap^TM^ FF crude column (Cytiva). His-tagged wt-M^pro^ and dm-M^pro^ were eluted from the column using a linear imidazole gradient (10–500 mM) and subsequently buffer-exchanged into buffer A. After overnight incubation at 12 °C with SUMO protease (ULP-1), cleaved wt-M^pro^ and dm-M^pro^ were purified by loading the sample onto a 1 ml Ni^2+^-HisTrap^™^ FF crude column equilibrated in buffer A and collecting the flow-through. As a final purification step, size-exclusion chromatography was performed using a Superdex 200 prep grade 16/600 SEC column (GE Healthcare) equilibrated in buffer C (20 mM Tris, 150 mM NaCl, pH 7.8). Protein identity and integrity were finally assessed by electrospray ionisation (ESI) mass spectrometry, which yielded experimental mass of 33797.0 Da for wt-M^pro^ (theoretical: 33796.6 Da) and 33699.1 Da for dm-M^pro^ (theoretical: 33698.5 Da).

### Inhibition of SARS-CoV-2 M^pro^ activity assay

The inhibitory activity of Ebselen and its analogues against SARS-CoV-2 wt-M^pro^ was evaluated *in vitro* using a fluorescence resonance energy transfer (FRET)-based proteolytic assay, by employing the fluorogenic substrate 5-FAM-AVLQ↓SGFRK(Dabcyl)K (Bachem, Bubendorf, Switzerland). The fluorescence of the intact peptide is very low since the fluorophore fluorescein (5-FAM) and the quencher Dabcyl are in close proximity. When the substrate is cleaved by the protease, the fluorophore and the quencher are separated, increasing the fluorescence signal. On the contrary, inhibition of M^pro^ results in a decrease of the fluorescence signal compared to the M^pro^ activity in the absence of an inhibitor. A preliminary screening was performed at a single compound concentration (first 100 µM and then 50 nM) to rapidly evaluate the compounds potential to inhibit M^pro^ activity and to estimate the range of inhibitory activity for subsequent dose-response analysis. The initial reaction velocity of 50 nM wt-M^pro^ was therefore measured at 37 °C in the absence (V_0_) and in the presence (V_i_) of increasing concentrations (0.5, 1, 2.5, 10, 50, 100, 500, 600 nM) of each compound of the library. Each reaction was initiated by adding 5 μM of fluorogenic substrate in reaction buffer (20 mM Tris, 100 mM NaCl, 1 mM EDTA, pH 7.4). For samples containing both the protease and the compound, a 20-min pre-incubation at room temperature was carried out immediately prior to initiating the reaction. Final v/v DMSO percentage was 3.75%. Fluorescence was measured using excitation and emission wavelengths of 485 nm and 535 nm, respectively, with a Victor3 microplate reader (PerkinElmer) for 50 min. The residual enzymatic activity of wt-M^pro^, expressed as the percentage ratio V_i_/V_0_, was plotted against each compound concentration, yielding the corresponding dose-response curve. IC_50_ values were finally fitted using GraphPad Prism 5 software and expressed as mean values ± standard deviation. Since this method relies on fluorescence detection, compounds with quenching properties could potentially interfere with the measurement, leading to false-positive results. To rule out this possibility, a counter-screening was conducted in parallel with the inhibition assays by monitoring the fluorescence emission of 0.5 μM free fluorescein in the absence and in the presence of each compound at concentrations ranging from 0.5 to 600 nM.

### Solvent-accessible surface areas (SASA) calculation

To calculate the solvent-accessible surface areas (SASA) of the cysteines in the free form of the symmetric M^pro^ dimer for wt-M^pro^ (PDB 6Y2E) and for the inactive double mutant His41Ala Cys145Ala (PDB 9EX8), we used the command-line tool FreeSASA, using default parameters and a spherical probe with a radius of 1.4 Å, approximating a water molecule^23^.

### Binding studies by mass spectrometry (MS)

Direct binding of each compound to the protein was investigated by electrospray ionisation mass spectrometry (ESI-MS) and by native mass spectrometry (nMS). All spectra were acquired in positive ion mode by using a Q-Tof Agilent 6550 iFunnel mass spectrometer (Agilent Technologies, California, USA) for denaturing conditions measurements and a Q-Tof Xevo G2S mass spectrometer (Waters, Manchester, UK) for native conditions analysis. All samples were prepared by mixing M^pro^ with a substoichiometric amount of each compound (final monomeric protein and compound concentrations of 5 μM and 2.5 μM, respectively) and incubated at room temperature for 20 min prior to analysis. For measurements under denaturing conditions, each mixture was in 20 mM Tris, 150 mM NaCl, pH 7 and contained 3.75% (v/v) DMSO. Samples were directly injected into the spectrometer using a flow rate of 100 μL/min with a 50:50 water/acetonitrile solution containing 0.1% formic acid. The resulting mass spectra were analysed and deconvoluted using the Agilent MassHunter BioConfirm 10.0 software. For the sake of clarity, the formation of covalent adducts between the wild-type or double-mutant form of M^pro^ and intact Ebselen or its derivatives was indicated in the mass spectra as the nominal mass of the tested compounds. For nMS analysis, samples were in 150 mM ammonium acetate, pH 7 to minimise the presence of salts that could interfere with MS measurements. The final DMSO concentration was maintained at 3.75% (v/v). The analysis was performed in nanoflow mode by using quartz emitters produced in-house by using a Sutter Instruments Co. (Novato, CA, USA) P2000 laser pipette puller. Up to 5.0 μL samples were typically loaded onto each emitter by using a gel-loader pipette tip. A stainless-steel wire was inserted into the back end of the emitter to supply an ionising voltage in the range of 1–1.5 kV. The source temperature was set at 30 °C. All experiments were performed in positive ion mode. Data were processed using Mass Lynx (v 4.2, SCN781; Waters) software. To evaluate the relative percentage of dimeric and monomeric wt-M^pro^ in each experiment, abundances of both protein-free and compound-bound M^pro^ species were calculated aided by in-house developed MATLAB (R2016b) scripts, expressed as a percentage and compared.

### Cell culture and virus

Vero E6 (ATCC^®^ CRL-1586TM) cells were grown in Dulbecco’s modified Eagle medium (DMEM; Thermo Fisher Scientific, Italy) supplemented with 10% (v/v) heat-inactivated foetal bovine serum (FBSi; Thermo Fisher Scientific). Cell cultures were maintained at 37 °C and 5% CO_2_ in 95% humidified atmosphere and regularly tested for mycoplasma contamination. Wuhan-like Italian clinical isolate SARS-CoV-2/human/ITA/CLIMVIB2/2020 (Genbank: MW000351) was selected for antiviral experiments.

### Antiviral activity in cell-based assays

Antiviral assays were conducted as previously described with few modifications^24^. Briefly, Vero E6 cells were seeded in 24-wells plate 24 h prior to infection, at a density of 1 x 10^5^ cells/well. The cell culture medium was removed and replaced with virus inoculum (cells were infected with 80 plaque-forming units -PFU- per well). After 1 h of incubation at 37 °C, the virus inoculum was removed, and cells were overlaid with 500 μL of 0.6% carboxymethylcellulose (Merck, Italy) diluted in DMEM supplemented with 2% FBSi and with the tested compounds (at different concentrations from 0.39 to 50 μM) or the vehicle (DMSO). Seventy-two hours post-infection, cells were fixed adding 500 μL of 5% formaldehyde (Merck, Italy) in PBS 1X for 30 min at room temperature. Then, cells were stained with 0.1% crystal violet in 20% ethanol. Plaques formed in the cell monolayer upon infection were counted. The antiviral activity for each concentration of the tested compounds was expressed as percentage in comparison to the control (untreated) cells.

All studies with SARS-CoV-2 were performed in the certified Biosafety Level-3 (BSL-3) laboratory of the Molecular Medicine Department, University of Padova.

## Results and discussion

### Ebselen derivatives are potent SARS-CoV-2 M^pro^ in vitro inhibitors

To assess the inhibitory potential of the new ebselen derivatives (Figure 1) against SARS-CoV-2 M^pro^ (hereafter referred to as wild-type or wt-M^pro^), we performed an *in vitro* fluorescence-based FRET inhibition assay. In a preliminary screening, all the compounds of series **1** and the N-acyl-derivatives of series **2**, were found to reduce the enzymatic activity of the viral protease by more than 50% already at nanomolar concentration (see Materials and Methods section). Enzymatic assays were therefore performed by titrating each compound in the range 0.5–600 nM. The resulting dose-response curves (Figure S1**)** were used to calculate the half-maximal inhibitory concentration (IC_50_) shown in Table 1, revealing that all tested compounds display IC_50_ values with single- and double-digit nanomolar values (Table 1).

**Table 1.** Inhibitory potency of Ebselen and its analogues against wt-M^pro^, expressed as IC_50_.

Compound	IC_50_ (nM)
1a (Ebselen)	8.35 ± 0.79
2b	7.20 ± 0.74
2d	8.66 ± 0.50
1b	8.69 ± 1.48
2g	8.83 ± 0.14
1c	9.79 ± 1.09
2c	9.98 ± 0.33
2f	11.01 ± 0.37
2e	13.44 ± 1.85
2a	45.00 ± 0.72

The compounds are ordered top to bottom, by decreasing inhibitory potency against wt-M^pro^. The IC_50_ values are reported as the mean ± standard deviation.

Under our experimental conditions, the positive control **1a** (hereafter simply Ebselen) inhibited wt-M^pro^ with an IC_50_ of 8.35 nM, on the lower edge of what reported in literature (30 nM)^25^. In the dose-response titrations, the cyclopropyl-substituted ureido derivative **2b** emerged as the most potent inhibitor exhibiting an IC_50_ of 7.20 nM. The cyclopropyl-substituted N-acyl-derivative **2c**, which had been indicated among the most promising derivatives by the testing for human neutrophil elastase inhibition and antiradical activity^21^, also showed single-digit nanomolar inhibition, while compound **2a**, the N-acyl-derivative bearing an ester moiety, was the weakest inhibitor of the series showing an IC_50_ of 45.00 nM. Notably, compound **1b**, which corresponds to the benzoselenazolone ring unit of Ebselen without the phenyl substituent, maintained a remarkable ability to suppress wt-M^pro^ activity (IC_50_ of 8.69 nM), indicating that the electrophilic selenium-containing warhead constitutes the minimal pharmacophore needed to efficiently inhibit the viral protease.

### Ebselen derivatives bind covalently to solvent accessible cysteines of SARS-CoV-2 M^pro^

**Scheme 1.**
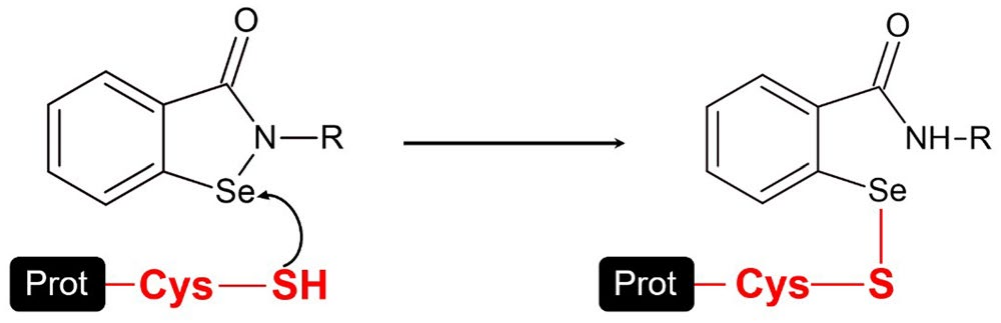
Reaction of Ebselen with the cysteine residues of proteins. The formation of the S–Se covalent bond results from the nucleophilic attack of a cysteine thiol group on the electrophilic selenium atom of the compounds. (R = Ph).

Ebselen has been employed in denaturing electrospray ionisation mass spectrometry (ESI-MS) experiments to label biological thiols in proteins and peptides by reacting covalently with cysteines (Scheme 1)^26^. Prompted by the high activity demonstrated by the tested compounds, we proceeded to determine whether the new derivatives bind to the wild type M^pro^ as Ebselen, which has been demonstrated to react covalently with thiol groups on cysteine residues within the protease^19^. To this aim, we evaluated the interaction with M^pro^ at the molecular level by ESI-MS, a technique which retains only covalent interactions between ligand and M^pro^^27^. Furthermore, since in the presence of a large molar excess of Ebselen all cysteine residues of wt-M^pro^ can be covalently modified^28^, we employed in our analysis a 0.5:1 compound-to-protein molar ratio to preferentially detect covalent modifications at the most reactive and/or more solvent-exposed cysteine residues of wt-M^pro^.

**Figure 2.**
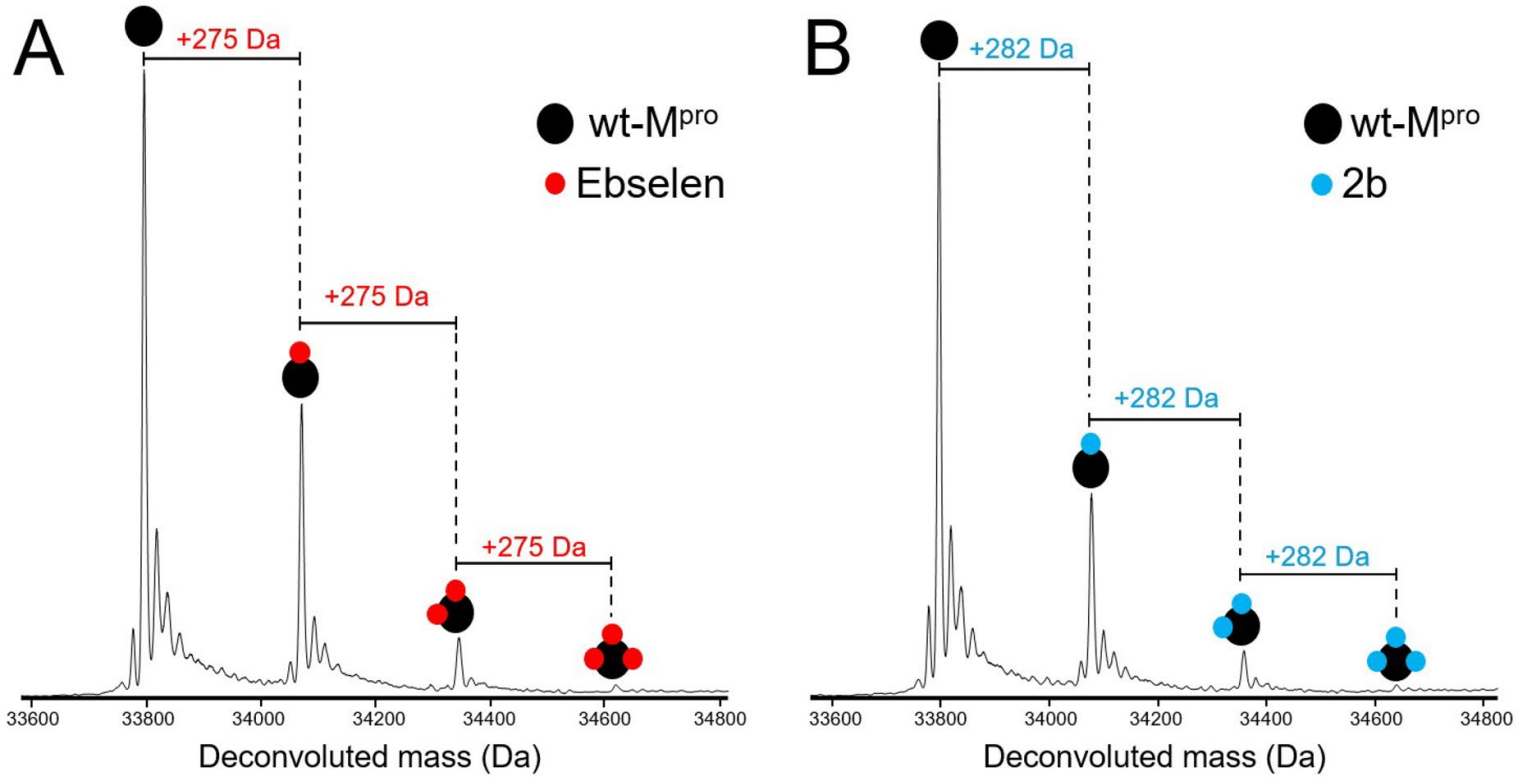
Deconvoluted ESI-MS spectra of 5 μM wt-M^pro^ (monomer concentration) treated with Ebselen (A) and compound 2b (B), by using a compound-to-protein molar ratio of 0.5:1 (i.e., 2.5 μM compound). Each spectrum was acquired in positive ion mode and revealed the presence of the unmodified protein and other three additional species corresponding to the 1:1, 2:1 and 3:1 compound:protein covalent adducts between monomeric wt-M^pro^ and Ebselen (mass shifts of + 275 Da, panel A) or compound 2b (mass shifts of + 282 Da, panel B).

ESI-MS analysis of mixtures containing wt-M^pro^ incubated with the Ebselen control revealed the presence of the unmodified protein along with three additional species exhibiting sequential mass increments of + 275 Da over the mass of the initial protein: these increments correspond to the masses expected for the monomeric wt-M^pro^ covalently bound by up to three Ebselen molecules (Figure 2A). Therefore, in the conditions of the ESI-MS analysis and at a limiting molar ratio, Ebselen retains its capability of covalently modifying the protein, likely through the formation of Se–S bond with the thiol group of multiple solvent-accessible cysteine residues. ESI-MS spectra were then recorded for **2b** (Figure 2B) and for all the new derivatives (Figure S2), showing in all cases incremental masses corresponding to different equivalents of covalently bound molecules. The mass spectrum recorded for the mixture containing wt-M^pro^ and compound **2b** displayed the pattern observed with Ebselen. In fact, we readily identified the unmodified monomeric protein and other three species with stepwise incremental mass of + 282 Da over the initial monomeric wt-M^pro^ (Figure 2B), corresponding to up to three equivalents of **2b** covalently bound to monomeric wt-M^pro^.

ESI-MS spectra recorded for the other ebselen derivatives (Figure S2) revealed that all compounds bind covalently to wt-M^pro^, with **1c** and **2c** also exhibiting up to 3 bound equivalents per protomer (Table S1). Interestingly, compound **1b**, corresponding to the benzoselenazolone scaffold, was the only one inducing up to 4 covalent species with wt-M^pro^ (Figure S2A and Table S1). Such high reactivity possibly reflects the lack of steric hindrance by substituents in the heterocycle ring of Ebselen. Thus, under our stringent concentration conditions, the reactivity was limited to a maximum of four covalent modifications per protomer, possibly corresponding to the most solvent-exposed or more reactive cysteines in wt-M^pro^. To assess the solvent exposure of cysteines in wt-M^pro^, we calculated their solvent-accessible surface area (SASA), a measure of the surface accessible to water molecules, using a spherical probe with the standard radius of 1.4 Å. As shown in Figure S3 (black bars), the SASA values for the 12 cysteine residues in each protomer of wt-M^pro^ indicate that cysteines 85, 145 (i.e., the catalytic one), 156, and 300 are the four most solvent-exposed and, based on that, the sites most likely targeted by Ebselen and its derivatives in our experimental conditions. However, we cannot exclude the possibility that wt-M^pro^ modified species include a low percentage of covalent adducts at C22 and C44, less solvent-exposed cysteine residues but predicted to be highly reactive^29^^,^^30^.

### Covalent binding of Ebselen derivatives to catalytically inactive SARS-CoV-2 M^pro^

**Figure 3.**
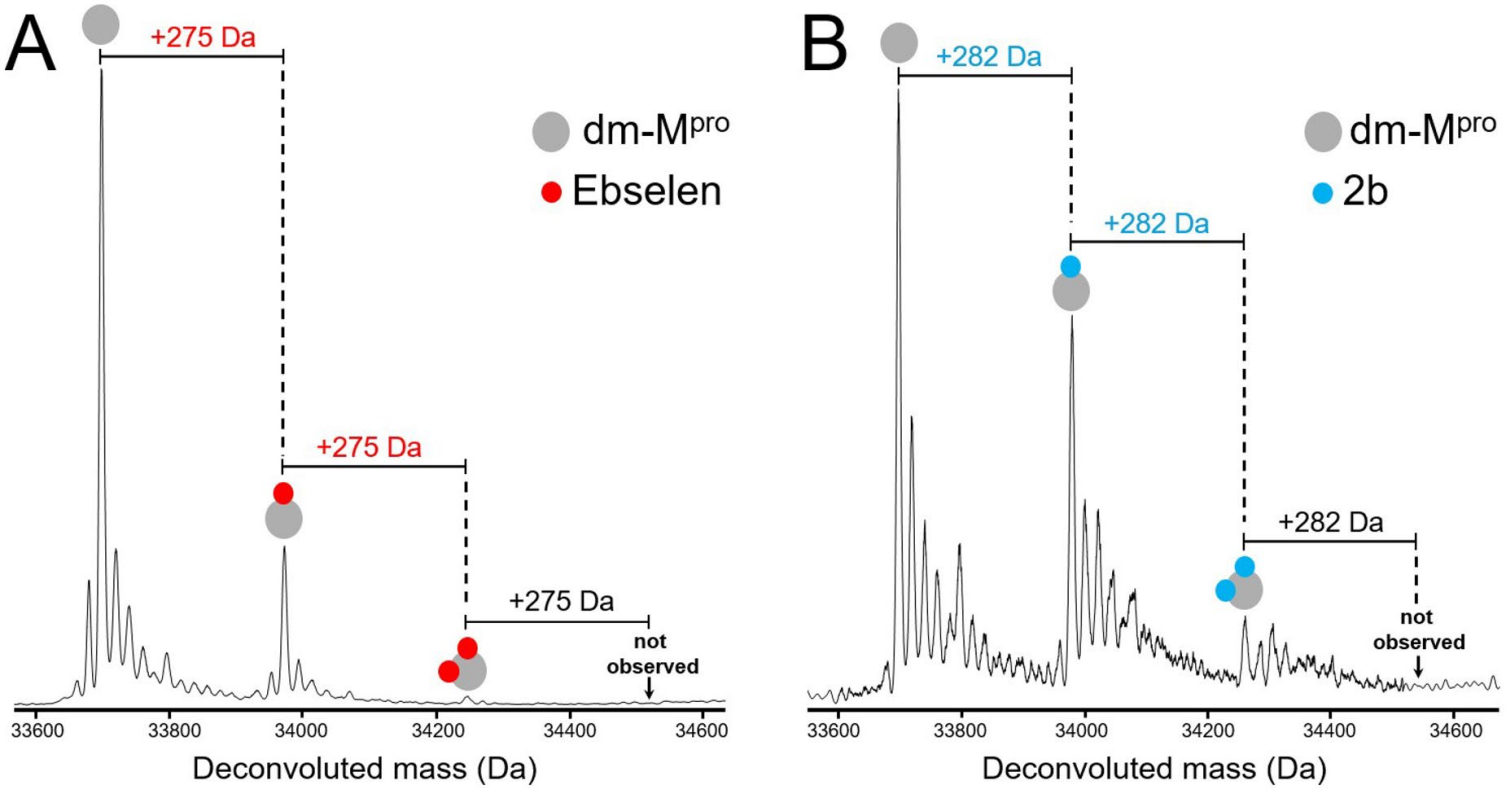
Deconvoluted ESI-MS spectra of 5 μM dm-M^pro^ (monomer concentration) treated with Ebselen (A) and compound 2b (B), by using a compound-to-protein-molar ratio of 0.5:1 (i.e., 2.5 μM compound). Each spectrum was acquired in positive ion mode and revealed the presence of the unmodified protein and other two additional species corresponding to the 1:1 and 2:1 compound:protein covalent adducts between monomeric dm-M^pro^ and Ebselen (mass shifts of + 275 Da, panel A) or compound 2b (mass shifts of + 282 Da, panel B).

The results described above do not provide direct evidence that the catalytic cysteine C145 is among the covalently modified residues of wt-M^pro^. ESI-MS analysis was therefore repeated at the same conditions above using a catalytically inactive mutant of the protease, in which both residues of the catalytic dyad, H41 and C145, were substituted by alanine^31^. This mutant will hereafter be referred as M^pro^ double mutant (dm-M^pro^). Since accessible cysteine residues in dm-M^pro^ exhibit SASA values mainly comparable to those in the wt-M^pro^ (see Figure S3, grey bars), we assumed that the putative loss of a covalent modification (i.e., covalent adduct detected in ESI-MS denaturing conditions) between dm-M^pro^ and the compounds could reasonably be attributed to the absence of C145. We therefore analysed the interaction between Ebselen and derivatives with dm-M^pro^ and at compound-to-protein-molar ratio of 0.5:1. Figure 3A reveals up to 2 bound molecules of Ebselen, and the same behaviour was found for compound **2b** (Figure 3B). Comparing the results with wt-M^pro^, the loss of 1 equivalent of bound molecule in the mutant enzyme supports the hypothesis that the ureido derivative **2b**, like Ebselen, might covalently target the catalytic C145 of wt-M^pro^^19^. The loss of one bound equivalent was also observed when dm-M^pro^ was incubated with the structurally related ebselen analogues **1b** and **1c** and with the N-cyclopropyl-derivative **2c** (Table S1).

Intriguingly, the N-acyl-derivatives of series **2** bearing bulkier substituents (i.e., **2a**, **2d**, **2e**, and **2 g**) showed only 2 bound equivalents in the wild type and only 2 bound equivalents in the double mutant protein experiment, while **2f** showed 1 bound equivalent with both proteins as indicated in Table S1. For the latter compounds, under the limiting compound concentrations of the experiment the absence of C145 in dm-M^pro^ may be compensated by binding at other sites of the enzyme, likely determined by the nature of substituents, eventually leading to the reaction with other cysteines. This does not necessarily imply that the bulkier compounds are unable to target the catalytic cysteine in wt-M^pro^ as they indeed showed to be active (Table 1). However, the variation in the number of covalent binding events to the protein does not appear to correlate directly with the inhibitory potency of the compounds and may be due to several factors, including different reactivity towards cysteines, limited solubility in aqueous buffers and contribution of non-covalent binding as suggested by molecular dynamic studies^32^. Our results, therefore, urged us to assess the behaviour of Ebselen and its derivatives at the supramolecular level of protein assembly.

### Ebselen and its derivatives shift the dimerisation equilibrium of wt-m^pro^ towards the inactive monomeric form

To gain deeper insight into the molecular mechanism of action of our inhibitors, we analysed their interaction with wt-M^pro^ by native mass spectrometry (nMS), which preserves protein assembly thanks to mild, non-denaturing conditions. We could therefore straightforward identify the enzyme’s dimeric (active) and monomeric (inactive) forms, their corresponding compound-bound species, as well as any variation in the equilibrium distribution of the oligomeric states of the enzyme upon compound interaction.

**Figure 4.**
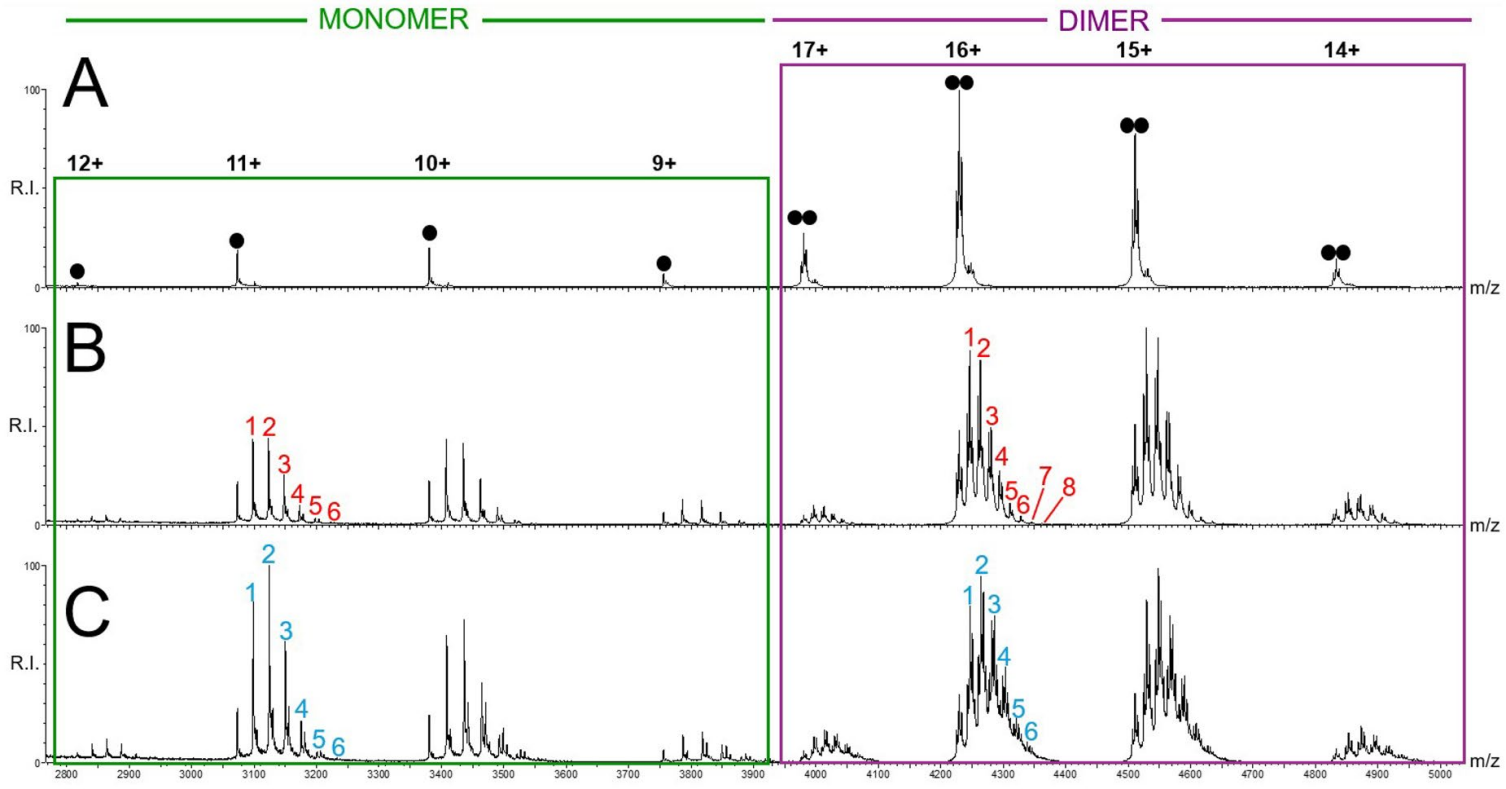
Representative native ESI-MS spectra of 5 μM wt-M^pro^ (monomer concentration) in the absence (A) and in the presence of Ebselen (B) and compound 2b (C), by employing a compound-to-protein-molar ratio of 0.5:1 (i.e., 2.5 μM compound). Spectra were recorded in positive ion mode in ammonium acetate 150 mM. The dimeric wt-M^pro^ is indicated by double black dots within the purple box, monomeric wt-M^pro^ is indicated by single black dots within the green box. In the presence of Ebselen (panel B) and compound 2b (panel C), the signals of monomeric and dimeric wt-M^pro^ exhibit sequential mass increments corresponding to the formation of binding complexes with multiple molecules of each compound. The number of interacting molecules is indicated by red numbers for Ebselen and cyan numbers for compound 2b; for the sake of clarity, numbers are indicated only for the 11+ and 16+ charge state for the monomer and the dimer, respectively.

An initial analysis with wt-M^pro^ alone revealed peaks with a well-resolved charge state distribution indicative of the enzyme dimer (Figure 4A). Minor peaks diagnostic of the monomer form confirm the coexistence of monomeric and dimeric states of the protein at 5 μM, consistent with the reported dissociation constant (0.14 μM) for the dimer–monomer equilibrium determined by previous native mass spectrometry studies^11^. We then analysed samples prepared by mixing wt-M^pro^ with each compound of the library, including Ebselen as control, maintaining the stringent compound-to-protein molar ratio of 0.5:1. The mass spectra recorded for wt-M^pro^ in the presence of Ebselen and compound **2b**, reported in Figure 4B and 4C respectively, clearly displayed additional peaks for both the monomeric and dimeric M^pro^. Specifically, the new species exhibited sequential mass shifts corresponding to +275 Da (Figure 4B) and +282 Da (Figure 4C) in the presence of Ebselen and **2b**, indicating that multiple molecules of both ligands bind to each of the two oligomeric forms of wt-M^pro^. The same analysis was performed for all ebselen derivatives (spectra reported in Figure S4) and revealed that all the compounds bind with multiple stoichiometries to both the dimeric and monomeric M^pro^.

**Figure 5.**
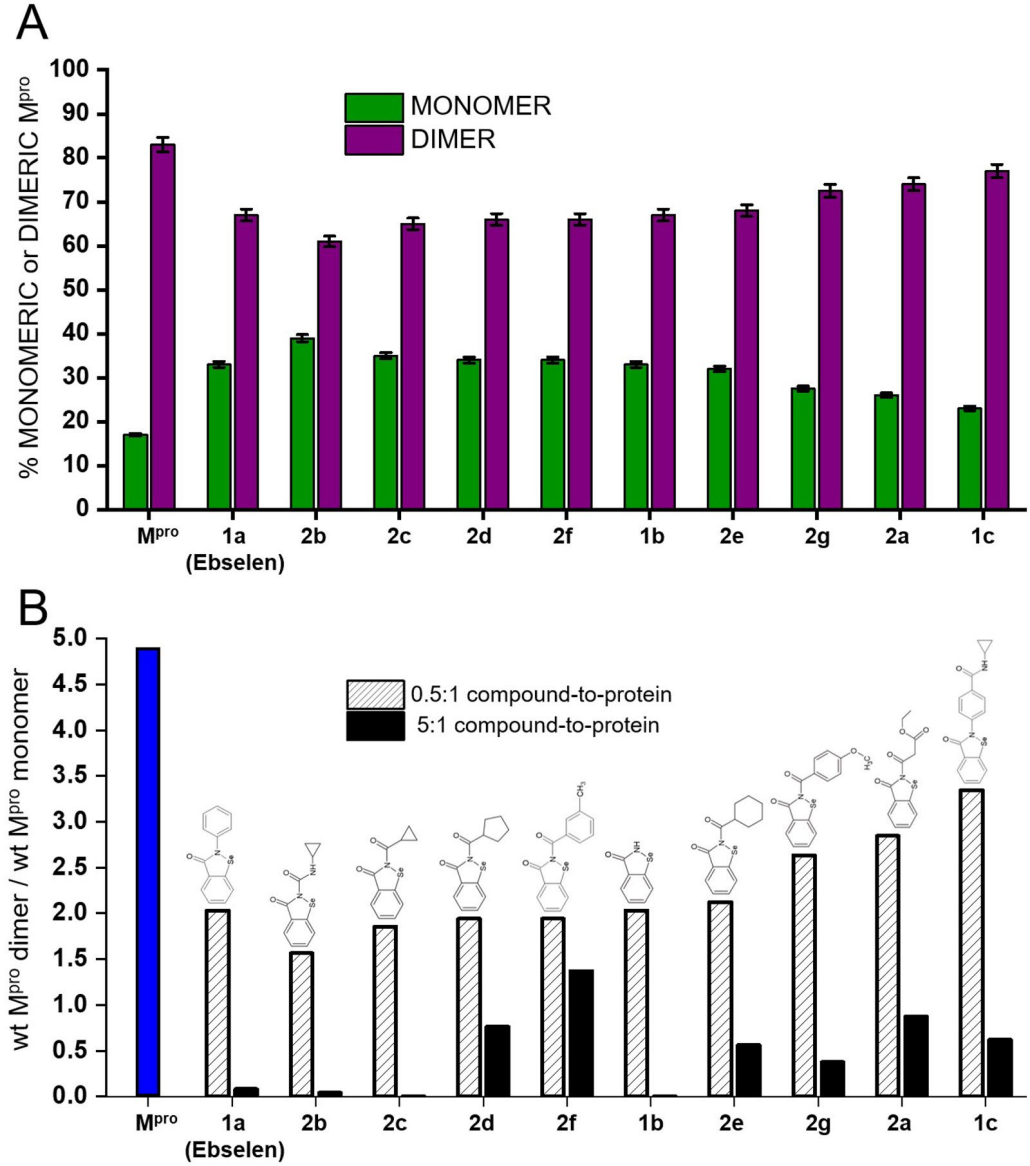
Quantitative analysis of the perturbation in the dimerisation equilibrium of wt-M^pro^ upon binding with Ebselen and its derivatives. (A) The histogram shows the relative percentages of wt-M^pro^ in its monomeric (green bars) and dimeric (purple bars) conformations, in the absence or presence of each compound of the library, as detected in the native mass spectra using a compound-to-protein molar ratio of 0.5:1. From left to right, ebselen derivatives are ordered according to their decreasing ability to shift the wt-M^pro^ dimerisation equilibrium towards the inactive monomeric form. (B) The histogram compares the ratio between dimeric and monomeric wt-M^pro^ in the absence (blue bar) or in the presence of Ebselen or its derivatives, using compound-to-protein molar ratio of 0.5:1 (dashed bars) and 5:1 (solid black bars). The relative percentages of wt-M^pro^ in its monomeric and dimeric conformations were calculated from native mass spectra of 5 μM wt-M^pro^ (monomer concentration) in the absence and presence of 2.5 μM compound (spectra reported in Figure 4 and S4, percentages in Figure 5(A)) and 25 μM compound (spectra and percentages reported in Figure S5).

Considering that the monomeric form of the protease is catalytically inactive^4^^,^^6^, we evaluated to which extent the treatment of wt-M^pro^ with Ebselen or any of its derivatives affected the dimerisation equilibrium of the protease. The relative abundances of dimeric and monomeric wt-M^pro^ in the absence of compound (spectra in Figure 4A) were used for the assessment of the equilibrium distribution, revealing 83% dimer and 17% monomer (Figure 5A). As expected under native conditions, the dimerisation equilibrium of the protease is strongly shifted towards its dimeric and active form, consistently with the reported equilibrium distribution of wt-M^pro^ (i.e., 89% dimer and 11% monomer)^11^. Low concentrations of Ebselen and derivatives had an evident effect on the dimerisation equilibrium of wt-M^pro^ as shown in Figure 5A: though the dimeric state of the protein remains the predominant form, the addition of substoichiometric ratio of all compounds revealed that the dimerisation equilibrium of wt-M^pro^ shifted towards the monomeric form of the protease, particularly in the case of compound **2b**, exhibiting a relevant increase of monomer up to 39% of total protein in solution. Figure 5B illustrates the equilibrium perturbation expressed as ratio of dimeric over monomeric wt-M^pro^ at different compounds’ concentration. At the lower concentration of compounds (2.5 μM, dashed bars in Figure 5B) the relative ratio of dimer over monomer was altered for all compounds, with the N-acyl-derivatives **2b** and **2c** significantly affecting the ratio dimeric/monomeric wt-M^pro^ (Figure 5B, dashed bars and Figure S4). We then tested the effect of excess compounds while maintaining constant the wt-M^pro^ concentration (i.e., compound-to-protein molar ratio 5:1). Native mass spectra of wt-M^pro^ in the presence of 25 μM Ebselen or derivatives (Figure S5) showed remarkable effects on the relative percentages of wt-M^pro^ dimer and monomer: a decrease in dimer (<10%), accompanied by a significant increase in inactive monomer (>90%), was observed for Ebselen, the unsubstituted **1b**, and for the cyclopropyl-substituted N-acyl-derivatives **2b** and **2c**. Figure 5(B) (solid black bars) quantifies the effects as ratio of dimeric over monomeric wt-M^pro^ at the higher concentrations of compounds: while the unsubstituted **1b** displayed a quite remarkable shift, in accordance with its higher accessibility to reactive sites of the enzyme in all our experimental conditions, the notable effects observed for **2b** and **2c** suggest that incorporating a cyclopropyl ring into the series of ebselen N-acyl-derivatives helps in tuning accessibility to sites of the enzyme conducive to destabilisation of the dimeric state of wt-M^pro^. Accordingly, adding a sizable cyclopropyl-amide moiety to the phenyl ring of Ebselen (**1c**), or modifying and increasing the size in the N-acyl derivatives (**2a, 2d-g**) led to a lower shift.

The observed changes of wt-M^pro^ dimerisation equilibrium might be due to their ability to bind accessible sites in the monomeric form of the protease, thus shifting the dynamic equilibrium of the enzyme towards the monomeric and catalytically ineffectual state. As shown in Figure S6, the calculated SASA of cysteine residues in wt-M^pro^ are largely conserved between the dimeric and monomeric forms of the protease, with two notable exceptions: C128 in domain II, which becomes only slightly accessible in the monomer, and C300 in domain III, whose accessible surface area nearly doubles in the monomeric state. Alternatively, direct binding at the wt-M^pro^ dimer interface could be responsible for perturbation of the dimeric state and subsequent increase in the monomer percentage. In both ways, the shift driven by Ebselen and related derivatives towards the monomeric form of the enzyme appears to be linked to the inhibitory activity, in accordance with what found in the case of glutathionylation of M^pro^^5^.

### Antiviral activity against SARS-CoV-2

Prompted by the significant impairment of SARS-CoV-2 M^pro^ dimerisation, we preliminarily evaluated the antiviral activity of the N-acyl-derivatives **2b** and **2c**, using Ebselen as control. Both Ebselen and **2c** induced a dose- dependent reduction within the range of 6–50 µM in viral infectivity on a plaque reduction assay (Figure S7), resulting in infectivity levels below 25% at the highest concentration tested. In contrast, compound **2b** exhibited limited effects, with infectivity reduction not exceeding 40%. The lower potency in the infected cell assay is attributed to limited membrane permeability of the two N-acyl-derivatives, as supported by the predicted logD values, with Ebselen exhibiting greater lipophilicity (logD = 2.65) than **2b** and **2c** (1.10 and 1.41, respectively), with the more hydrophilic compound **2b** showing poorer efficacy. The cyclopropyl-derivative **2c**, whose cytotoxicity has been shown to be lower than **2b**^21^, represents instead a hit to be developed not only as a dual anti-HNE and antiradical compound, but also as a potential drug for coronavirus diseases.

## Conclusions

Our study proves the hypothesis that dual antioxidant and anti-HNE compounds retaining the benzoselenazolone electrophilic warhead of Ebselen in their molecular structure are able to target the SARS-CoV-2 M^pro^ enzyme^21^. We report here that these compounds are potent *in vitro* inhibitors of the catalytic activity of the viral protease exhibiting potencies in the nanomolar range. As they all share the electrophilic benzoselenazolone core of Ebselen, we hypothesised that their mechanism of inhibition would occur via covalent modification of thiol groups on accessible cysteine residues within M^pro^. ESI-MS spectrometry showed covalent modifications of the enzyme with the addition of the whole compound mass with the open selenazolone ring as in Scheme 1, in accordance with other authors^19^^,^^28^, but in contrast with crystallographic investigations which had proposed the formation of an unstable adduct eventually released as free salicylanilide^17^.

As previously reported, Ebselen can covalently bind to various cysteines^19^^,^^28^^,^^33^. Accordingly, we found by ESI-MS that, besides Ebselen, the two derivatives with smaller N-acyl substituents (**2b** and **2c**) reacted with the enzyme forming covalent adducts with several cysteines, and the experiments with the inactive double mutant suggested that one covalent linkage had involved the catalytic C145. Since Ebselen and those derivatives behaving similarly bind not only to C145 but also to other cysteines, the amount of ligand capable of inhibiting M^pro^ through the catalytic site is lower than the theoretical one in solution. Hence, the observed inhibitory activity must be mediated not only by targeting the catalytic cysteine but also by other concomitant reactions.

Very interesting insights were indeed found by mass spectrometry experiments conducted under native conditions respectful of the protein assembly in its catalytically competent state. Ebselen induced an evident change in the dimer-monomer equilibria of M^pro^, and this behaviour was observed to varying degrees for all derivatives. The shift towards the monomer was augmented at higher compound concentration, with the least hindered compounds of the N-acyl series exhibiting quite dramatic effects. We have no evidence of the molecular events responsible for monomer population increase, although we trace it in the reactivity of benzoselenazolone electrophilic warhead with cysteines. It is tempting to think that, besides C145, the best candidate for the covalent binding could be C300, which, according to SASA values, is the most exposed cysteine in dimeric and in monomeric M^pro^. Actually, it has been shown that modification of C300 by glutathione leads to the destabilisation of the dimeric state of wt-M^pro^ thereby lowering enzymatic activity of the protease^5^. C300 is the cysteine in the C-terminal tail of the protein located at the interface between the two subunits, close to a hydrophobic pocket previously identified as a possible binding site of M^pro^ inhibitors^12^. The correct positioning of the C-terminal tail connecting the two subunits is important for the stabilisation of the dimer, as documented by the observation that mutation of arginine 298 to alanine destabilises the supramolecular protein assembly^6^. It is therefore likely that covalent modification of C300 by Ebselen and derivatives is involved into the observed shift towards the monomeric form. Notably, unlike other substrates or peptidomimetic inhibitors that, by targeting only C145, stabilise the dimeric form of the enzyme^6^^,^^31^^,^^34^, Ebselen and the N-acyl derivatives **2b** and **2c** that can simultaneously target several cysteines show the opposite effect stabilising the monomeric form. Although **2b**, our most potent enzyme inhibitor *in vitro*, was a valuable tool in mass spectrometry studies, its unfavourable LogD precluded antiviral effects in the tested system. The higher lipophilicity of the cyclopropyl-derivative **2c**, albeit lower than Ebselen, granted instead activity in infected cells and potential for development.

In conclusion, the mechanism of action of Ebselen and its derivatives is more complex than what reported for the vast majority of covalent M^pro^ inhibitors and our work indicates another strategy for designing new leads for drug discovery. As these compounds share similarities in their manifold behaviour towards M^pro^, we hope that our results will reinforce the interest towards Ebselen-derived inhibitors of coronavirus M^pro^.

## Supplementary Material

Fabbian_et_al_SUPPLEMENTARY.docx

## Data Availability

The data that support the findings of this study are available upon reasonable request.
