# Supplementary material for: Insights into the complexity of SARS-CoV-2 Mpro inhibition: Ebselen and its derivatives impair dimerisation of the enzyme
Source: J Enzyme Inhib Med Chem. 2026 Jan 2;41(1):2604232. doi: 10.1080/14756366.2025.2604232 (PMC12777896; doi:10.1080/14756366.2025.2604232)

**SUPPORTING INFORMATION**

**Insights into the complexity of SARS-CoV-2 M^pro^ inhibition: Ebselen and its derivatives impair dimerization of the enzyme**

**TABLE OF CONTENTS**

**Figure S1.** Dose-response curves resulting from the SARS-CoV-2 M^pro^ inhibition in the presence of Ebselen and its derivatives. **P.2**

**Figure S2.** Deconvoluted ESI-MS spectra under denaturing conditions of either wt-M^pro^ or dm-M^pro^ treated with ebselen derivatives. **P.3**

**Table S1.** Covalent adducts of Ebselen and its derivatives with wt-M^pro^ and dm-M^pro^ detected by ESI-MS. **P.6**

**Figure S3.** Comparison of the Solvent-Accessible Surface Area (SASA) of cysteine residues in SARS-CoV-2 wt-M^pro^ and dm-M^pro^. **P.8**

**Figure S4.** Representative native ESI-MS spectra of wt-M^pro^ in the presence of ebselen derivatives. **P.9**

**Figure S5.** Representative native ESI-MS spectra of wt-M^pro^ in the presence of 5-fold molar excess of Ebselen or its derivatives. **P.12**

**Figure S6.** Comparison of Solvent-Accessible Surface Area (SASA) of cysteine residues in each protomer of dimeric and monomeric SARS-CoV-2 wt-M^pro^. **P.15**

**Figure S7.** Antiviral activity of Ebselen and the acyl-derivatives 2b and 2c on Vero E6 cells infected with SARS-CoV-2. **P.16**

**Figure S1. Dose-response curves resulting from the SARS-CoV-2 M^pro^ inhibition in the presence of Ebselen and its derivatives.** Each dose-response curve represents the percentage of residual enzymatic activity of wt-M^pro^ (V%, plotted on the y-axis, blue curves) measured in the presence of increasing concentrations (0.5-600 nM) of Ebselen or the other tested compounds. A counter-screening control experiment (FAM fluorescence, black curves) was performed by using the same range of compound concentrations in the presence of 0.5 μM free fluorescein.

| 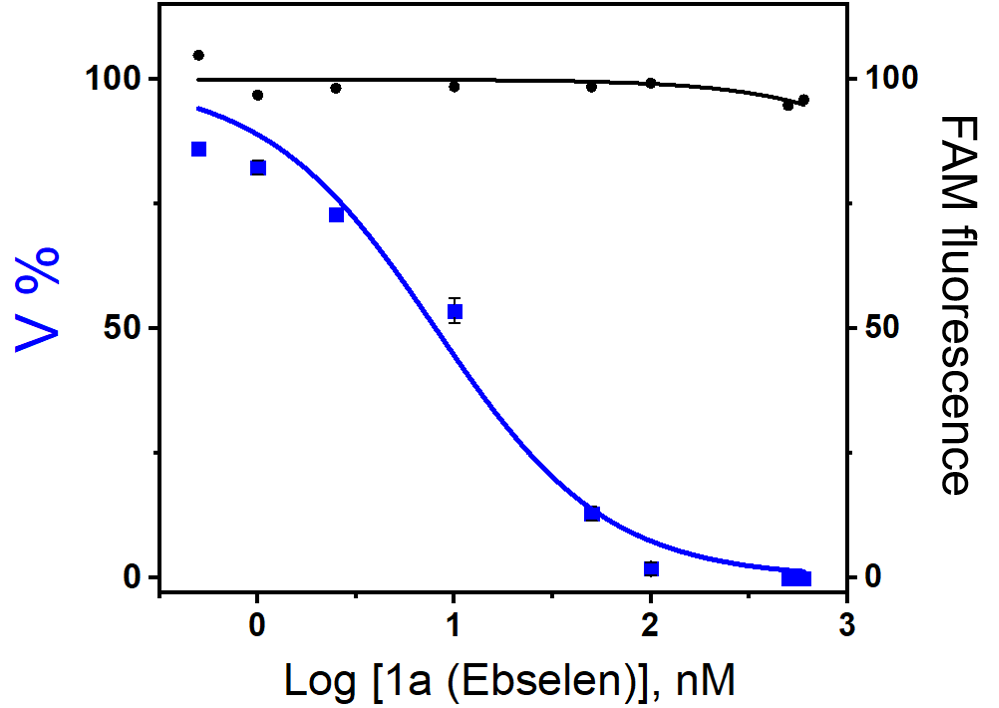 | 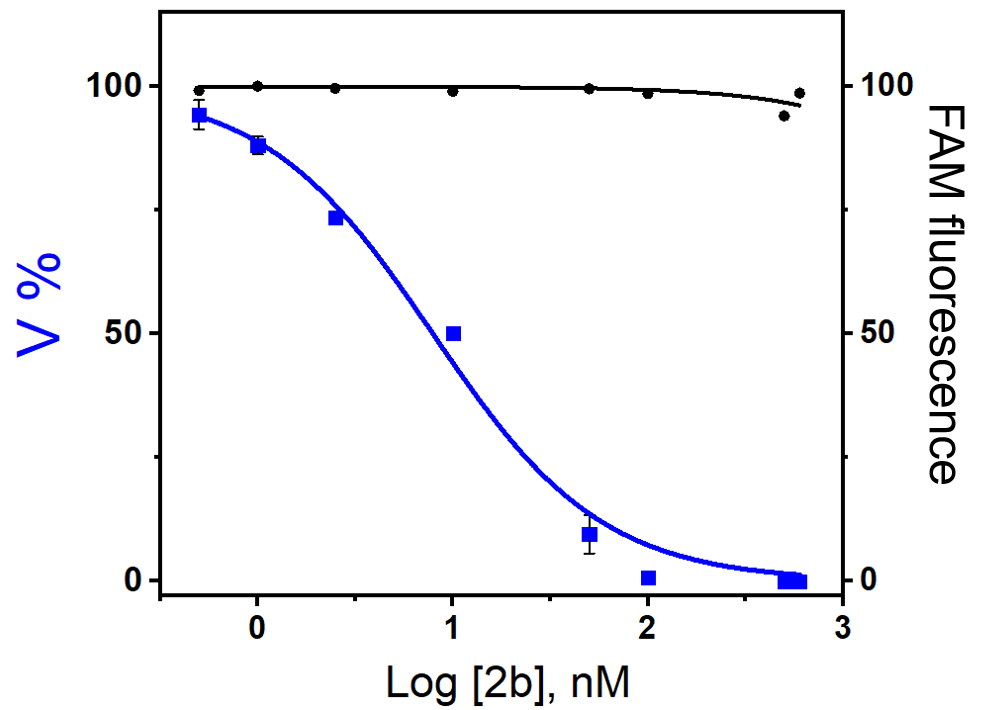 | 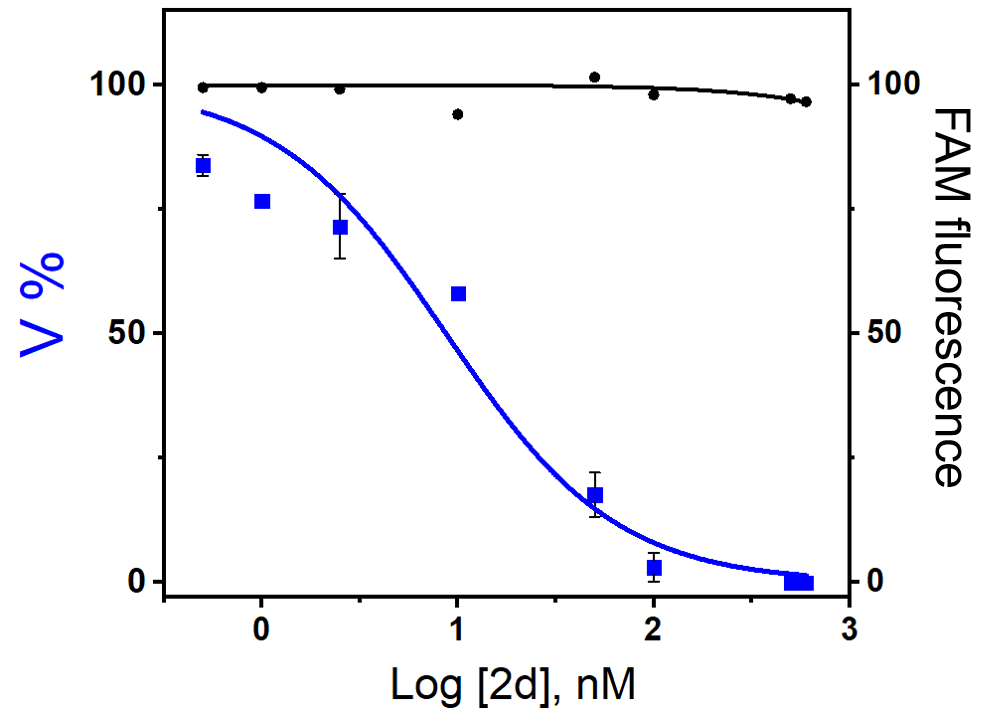 |
| --- | --- | --- |
| 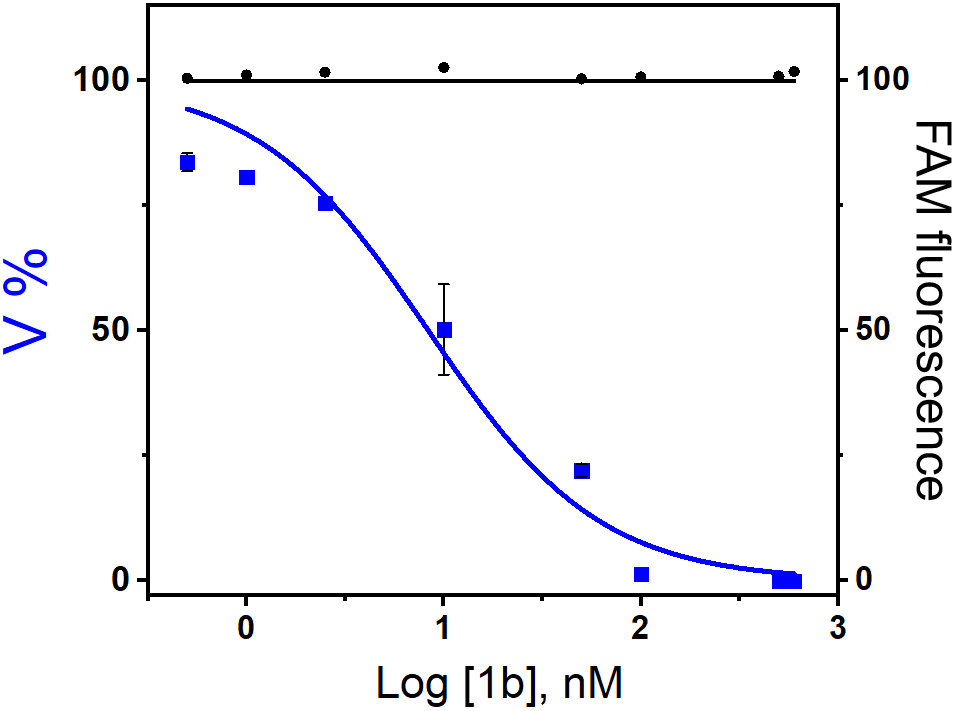 | 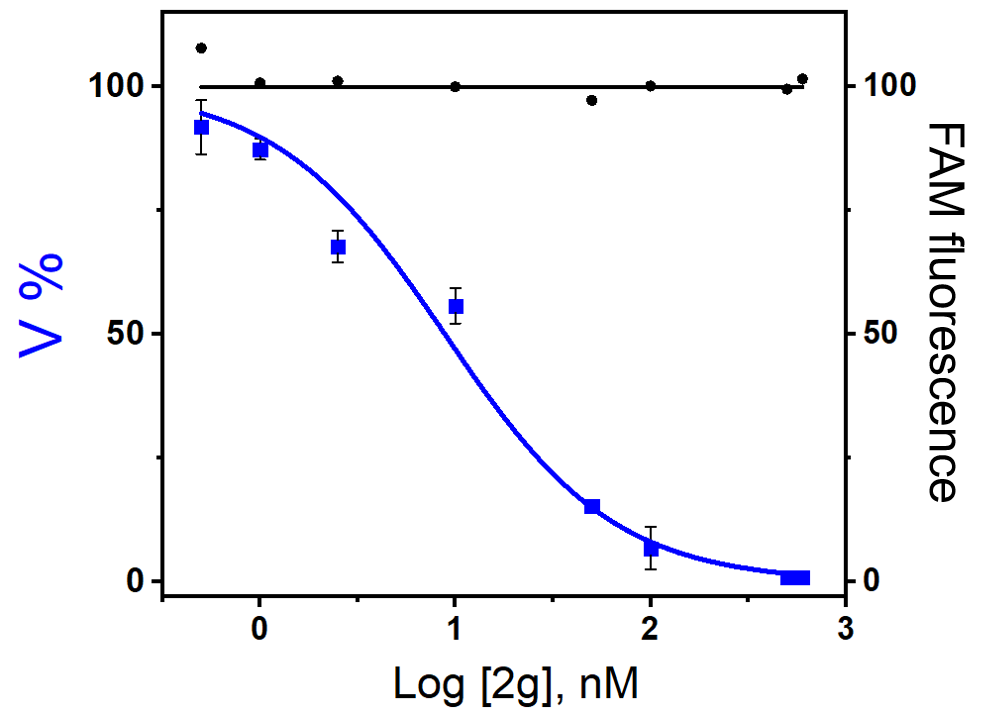 | 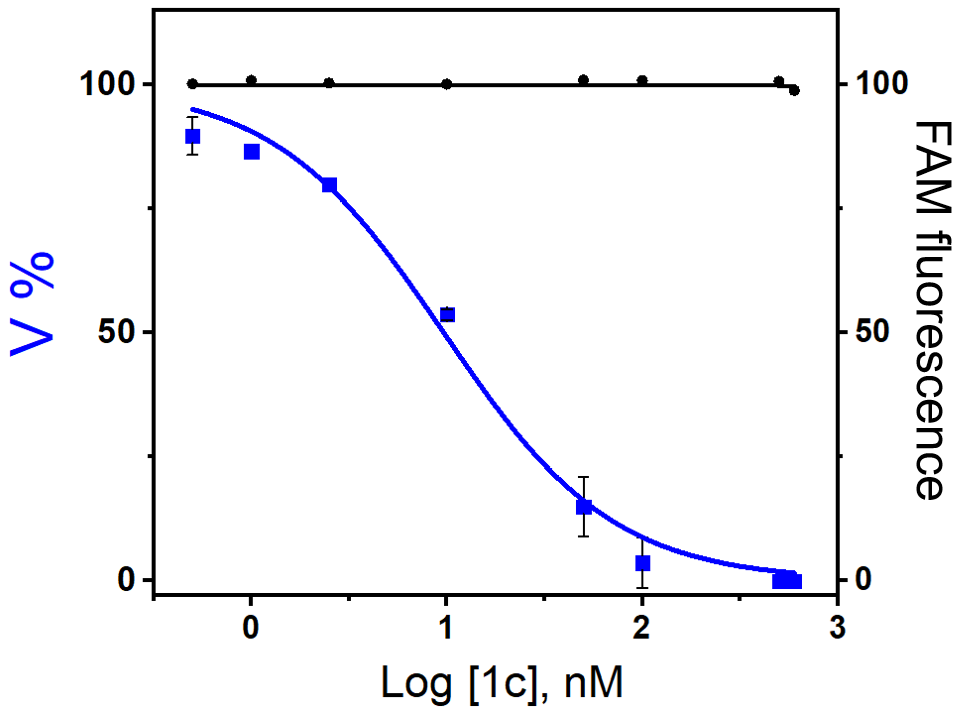 |
| 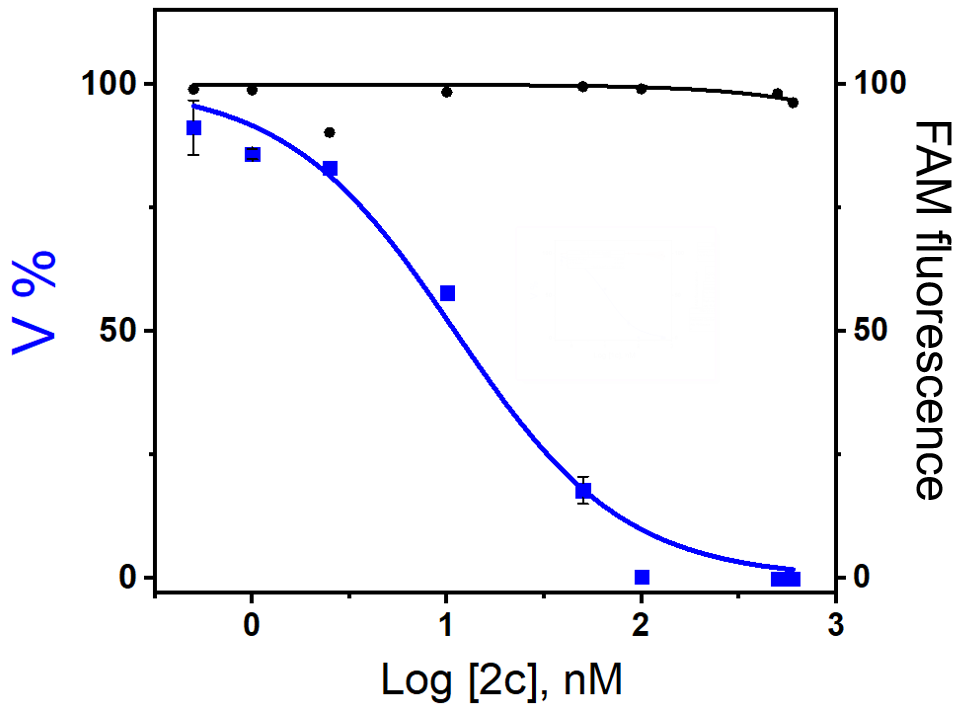 | 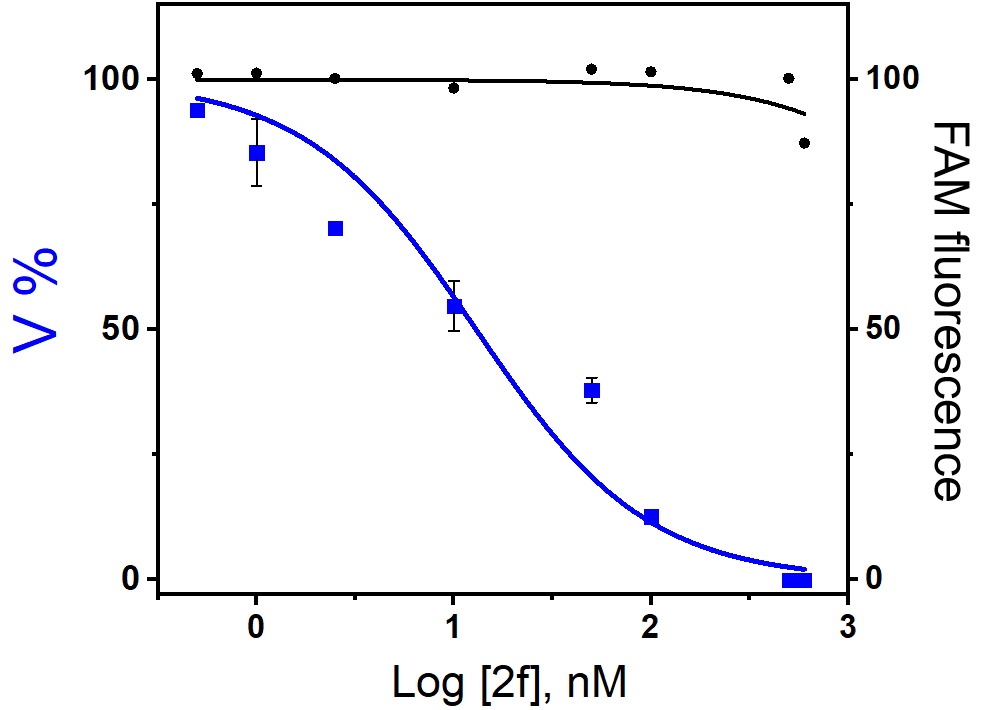 | 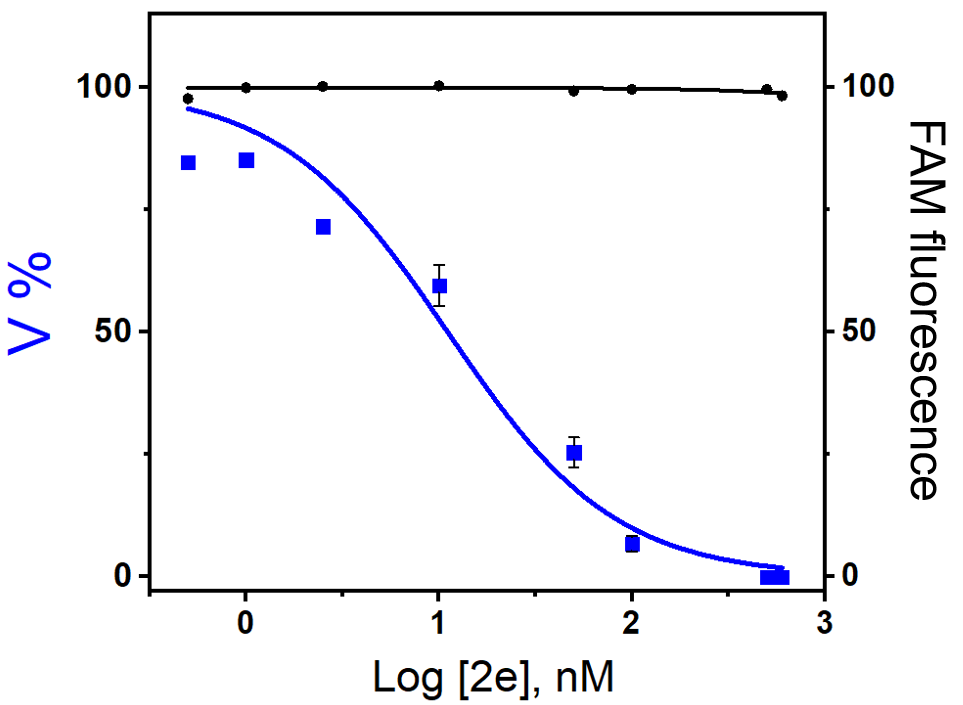 |
| 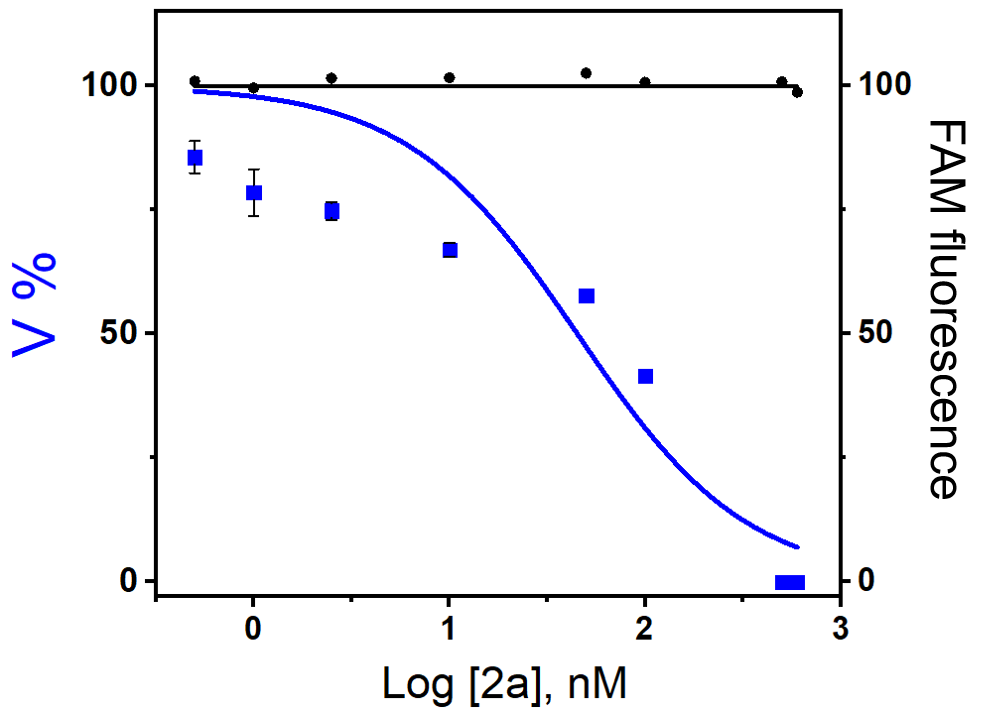 |  | |
|  | | |

**Figure S2. Deconvoluted ESI-MS spectra under denaturing conditions of either wt-M^pro^ or dm-M^pro^ treated with ebselen derivatives.** The analysis was performed using 5 μM wt-M^pro^ (monomer concentration) in the presence of 2.5 μM of compound (compound-to-protein molar ration of 0.5:1). Each spectrum was acquired in positive ion mode and revealed the presence of the unmodified protein along with additional species corresponding to covalent adducts between monomeric wt-M^pro^ or dm-M^pro^ and the tested compounds. The observed mass shifts relative to the unmodified proteins correspond to the molecular weights of the intact compounds. Panels (compound): **A**(**1b**), **B**(**1c**), **C**(**2c**), **D**(**2a**), **E**(**2d**), **F**(**2e**), **G**(**2g**) and **H**(**2f**).

| 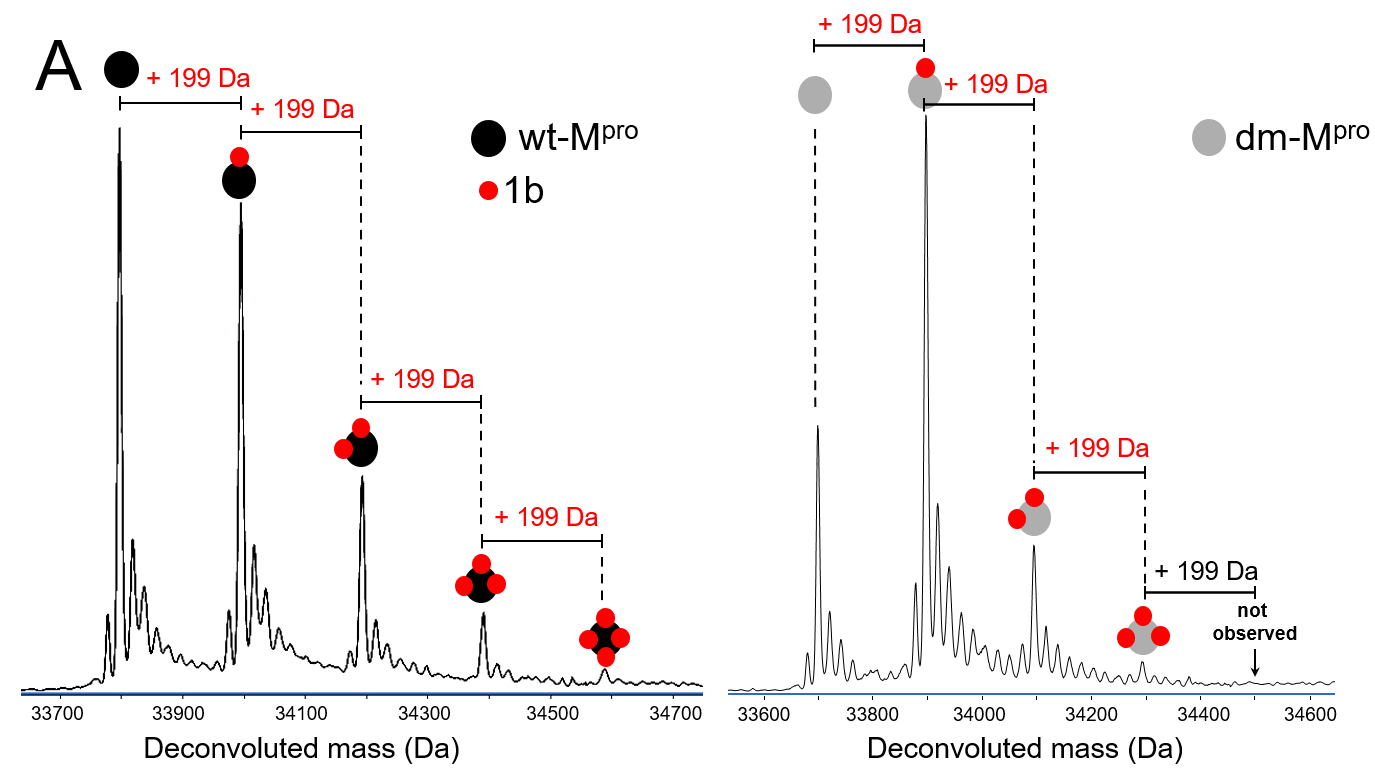 |
| --- |
| 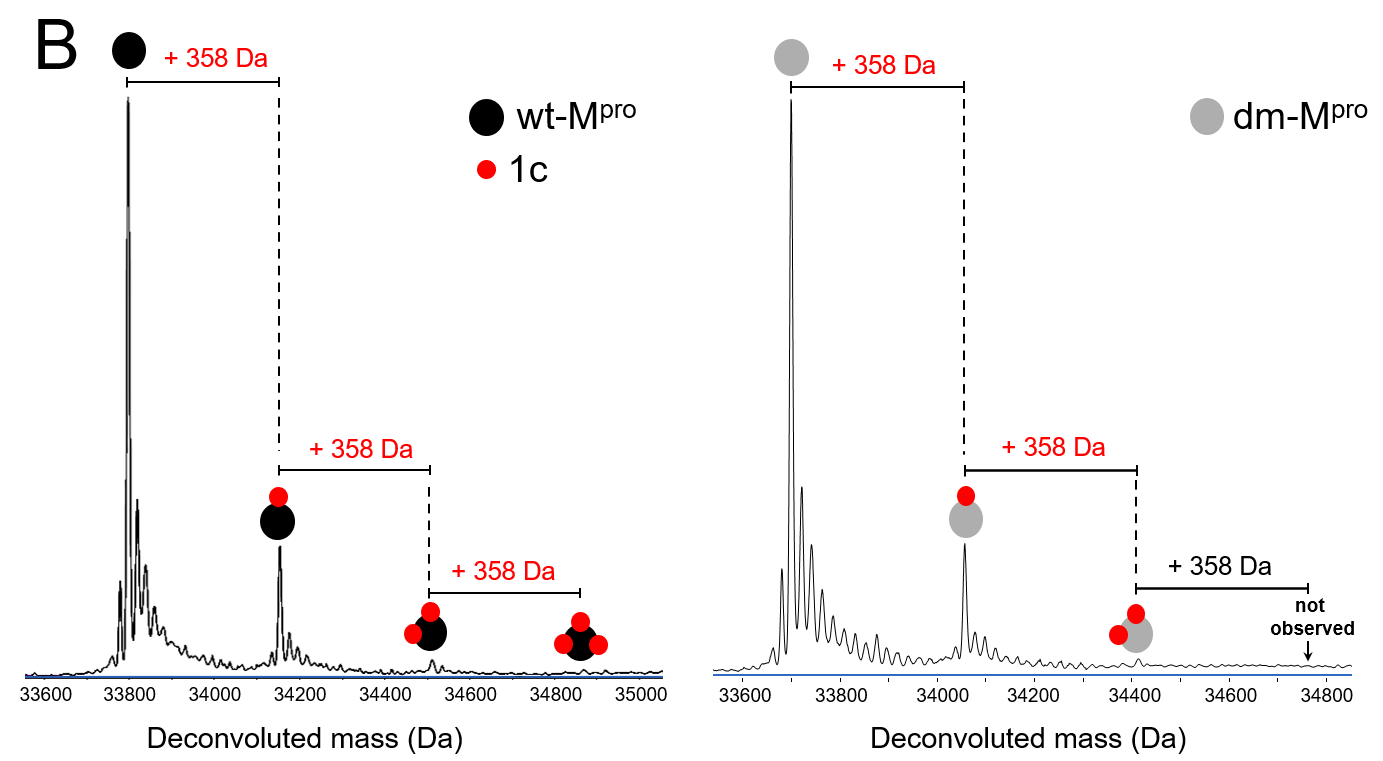 |
| 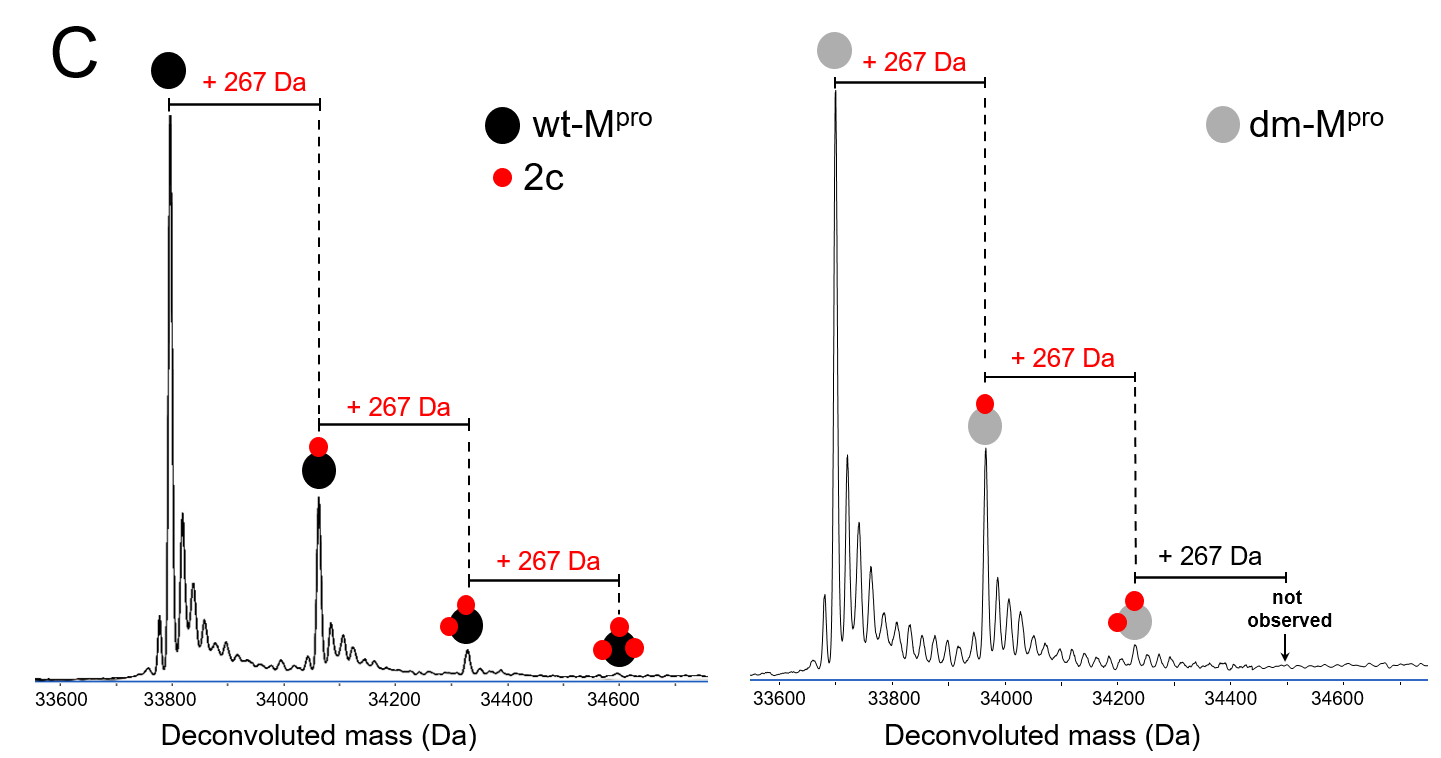 |
| 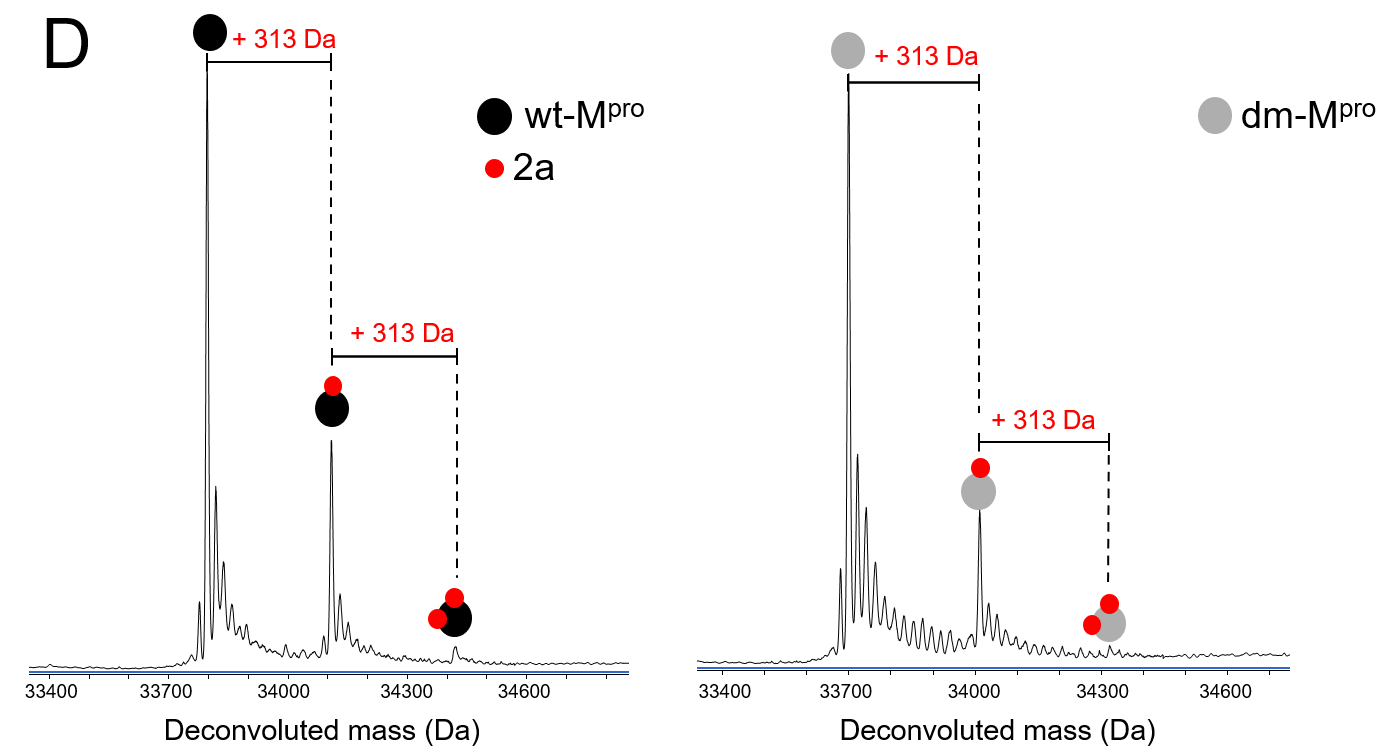 |
| 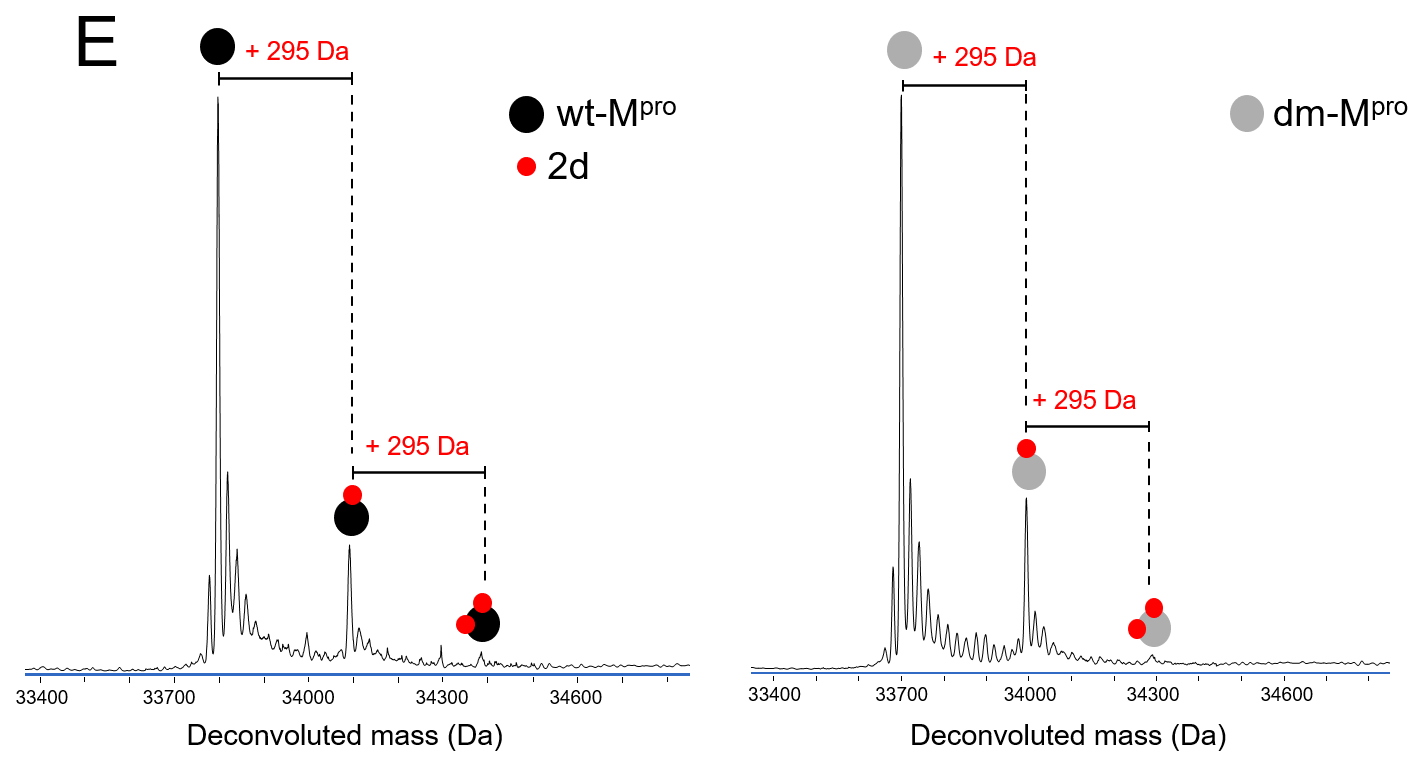 |
| 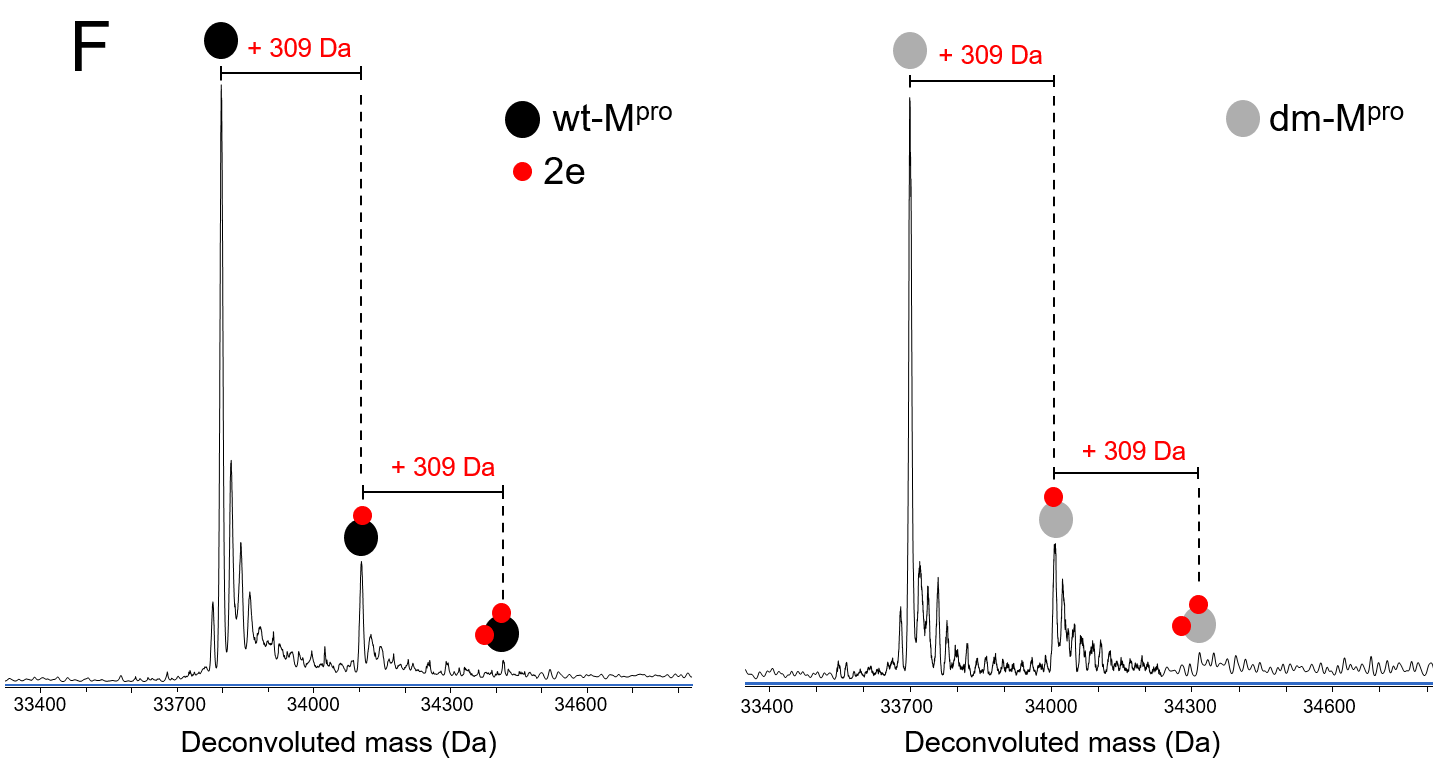 |
| 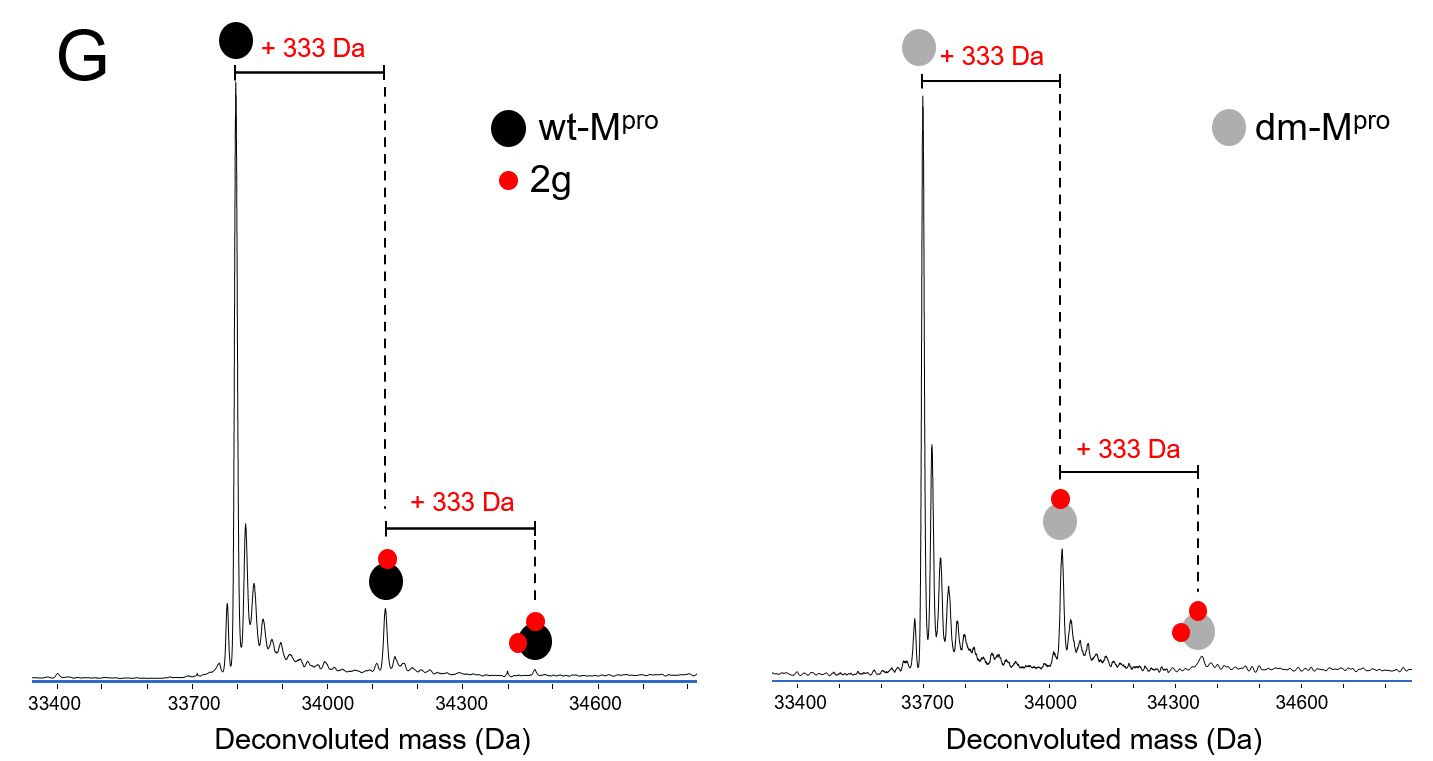 |
| 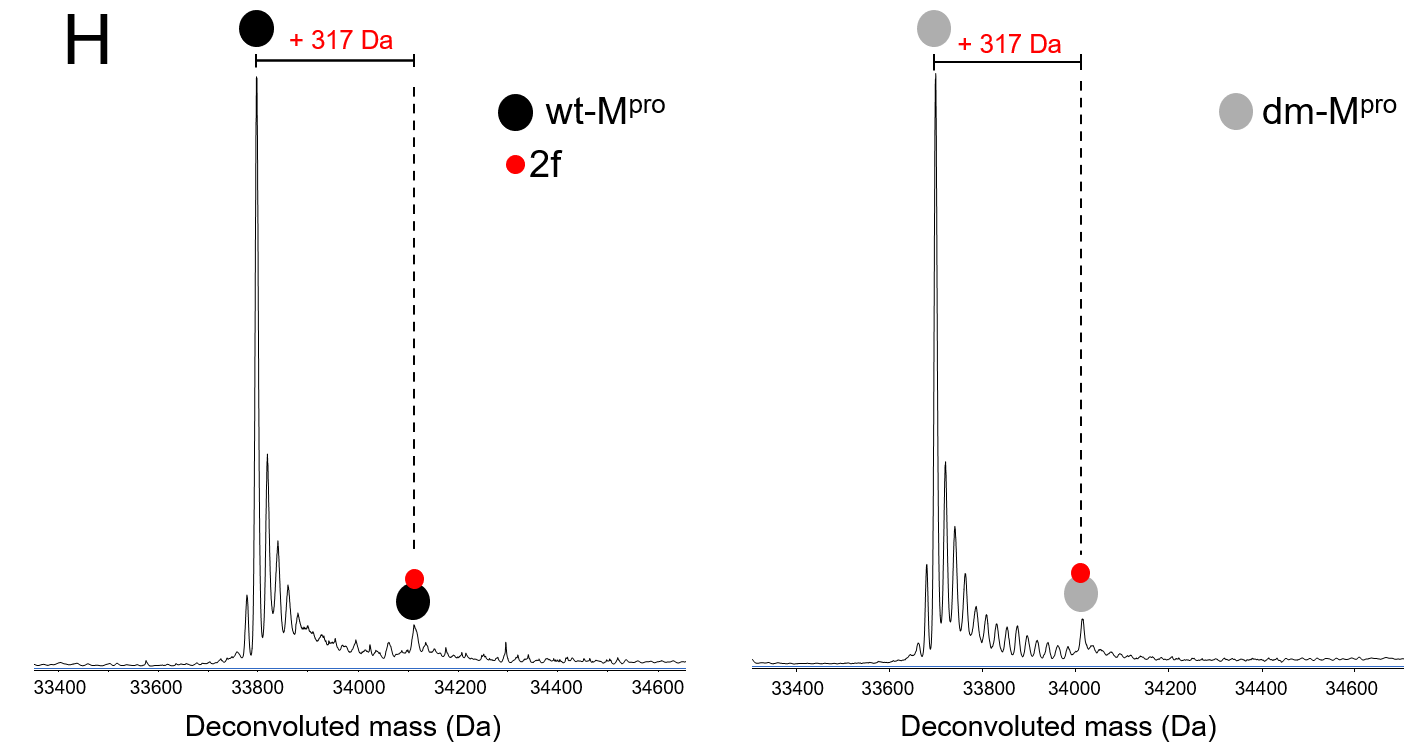 |

**Table S1. Covalent adducts of Ebselen and its derivatives with wt-M^pro^ and dm-M^pro^ detected by ESI-MS.** The analysis was performed as described in the caption of **Figure S2**. The number of ligand molecules covalently bound to the monomeric form of either wt-M^pro^ or dm-M^pro^ is reported for each tested compound. The difference (Δ) indicates the change in covalent interactions with dm-M^pro^ relative to wt-M^pro^.

| **Compound** | **Compound chemical structure** | **Equivalents of compound bound to monomeric wt-M^pro^** | **Equivalents of compound bound to monomeric dm-M^pro^** | **Difference (Δ) of covalent interactions of dm-M^pro^ relative to wt-M^pro^** |
| --- | --- | --- | --- | --- |
| **1a** (Ebselen) | 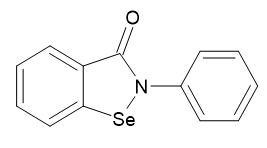 | 3 | 2 | −1 |
| **1b** | 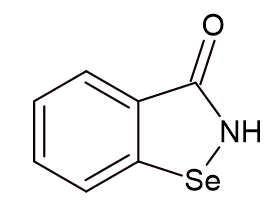 | 4 | 3 | −1 |
| **1c** | 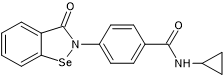 | 3 | 2 | −1 |
| **2b** | 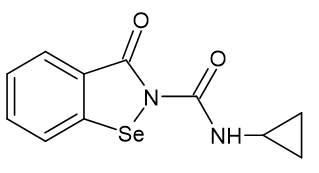 | 3 | 2 | −1 |
| **2c** | 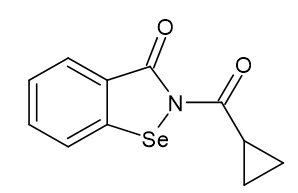 | 3 | 2 | −1 |
| **2a** | 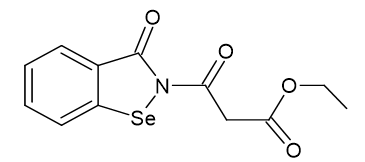 | 2 | 2 | 0 |
| **2d** | 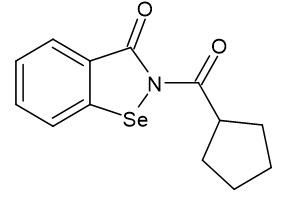 | 2 | 2 | 0 |
| **2e** | 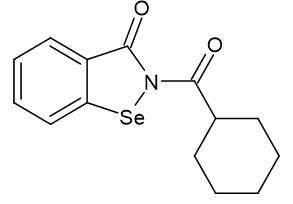 | 2 | 2 | 0 |
| **2g** | 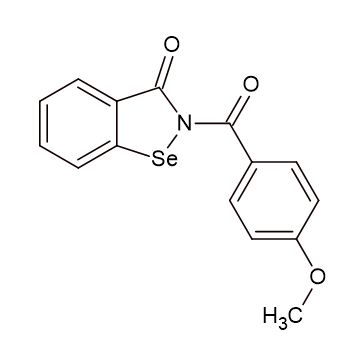 | 2 | 2 | 0 |
| **2f** | 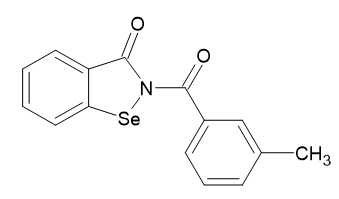 | 1 | 1 | 0 |

**Figure S3. Comparison of the Solvent-Accessible Surface Area (SASA) of cysteine residues in SARS-CoV-2 wt-M^pro^ and dm-M^pro^.** Solvent-Accessible Surface Areas (SASA, Å^2^) were calculated for the 12 cysteine (C) residues in a protomer of the free form of the symmetric dimer of SARS-CoV-2 wt-M^pro^ (black bars, PDB 6Y2E) and dm-M^pro^ (grey bars, PDB 9EX8) and their values are reported above each bar of the histogram. As the two protomers in the symmetric dimer are related by a perfect two-fold symmetry axis, their SASA values are identical. The structure of dimeric wt-M^pro^ (PDB 6Y2E) is shown above the histogram, with domains I (residues 10-99) in magenta, II (residues 100-182) in green and III (α-helical dimerization domain, residues 198-303) in cyan, depicted for one protomer. The 12 cysteine residues of this protomer are indicated by filled red circles. The second, symmetric protomer is coloured in black.

| 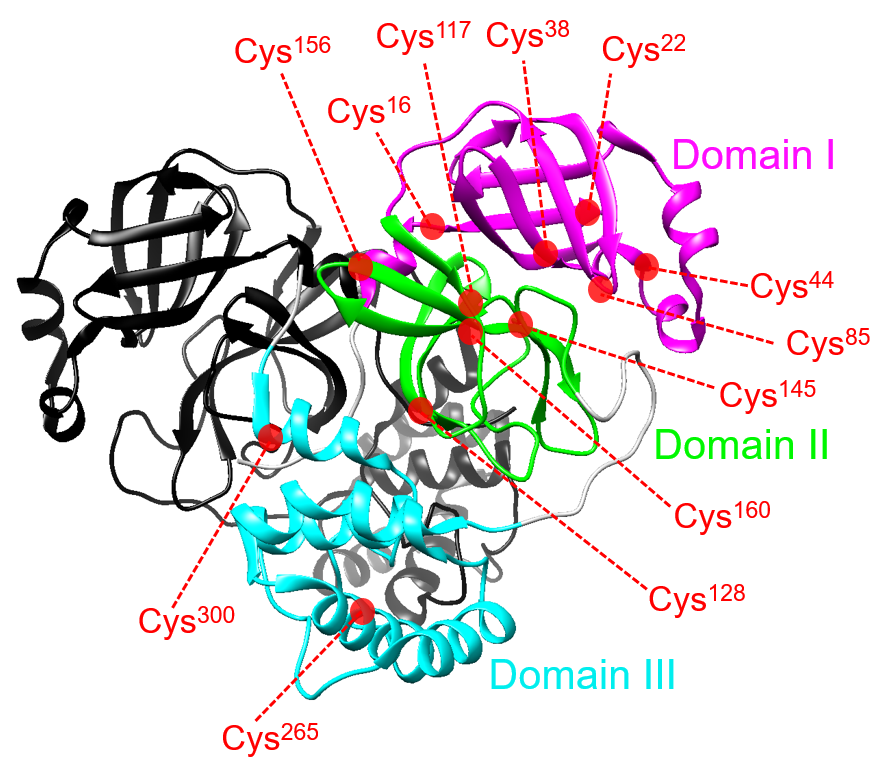 |
| --- |
| 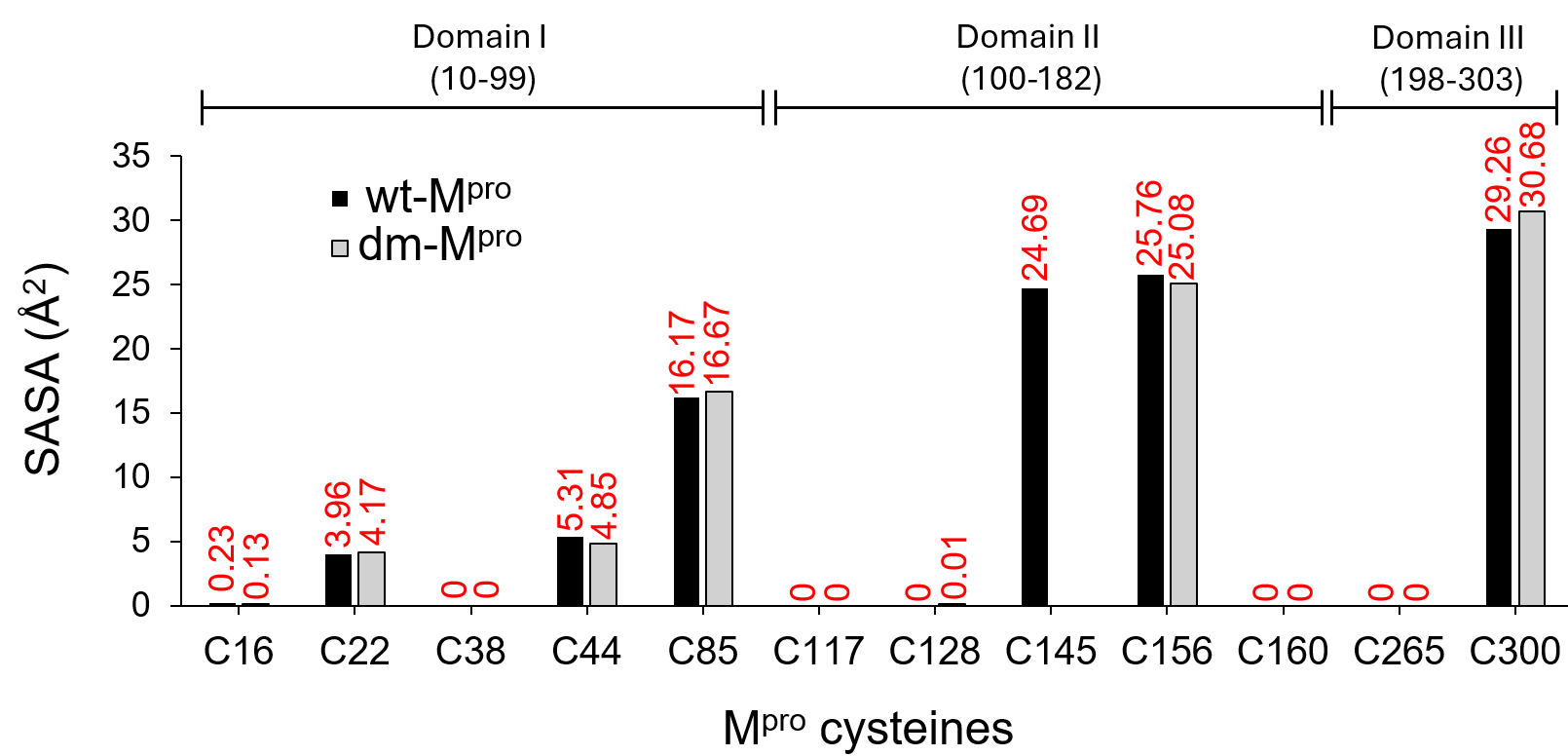 |

**Figure S4. Representative native ESI-MS spectra of wt-M^pro^ in the presence of a substoichiometric amount of ebselen derivatives.** The analysis was performed using 5 μM wt-M^pro^ (monomer concentration) in the presence of 2.5 μM of compound (compound-to-protein molar ration of 0.5:1). The signals of monomeric and dimeric wt-M^pro^ exhibit sequential mass increments corresponding to the formation of binding complexes with multiple molecules of each compound. Panels (compound): **A**(**1b**), **B**(**1c**), **C**(**2c**), **D**(**2a**), **E**(**2d**), **F**(**2e**), **G**(**2g**) and **H**(**2f**).

| 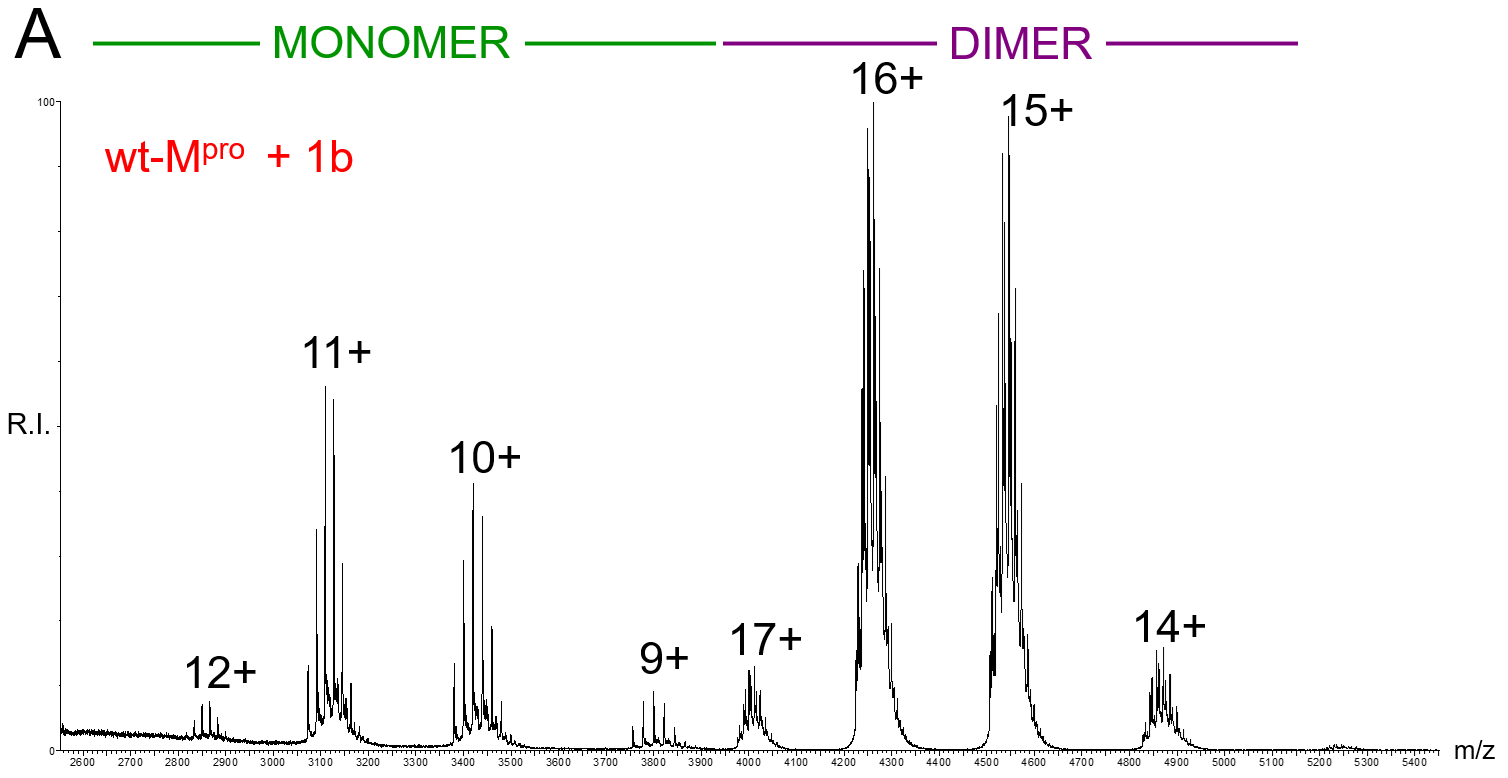 |
| --- |
| 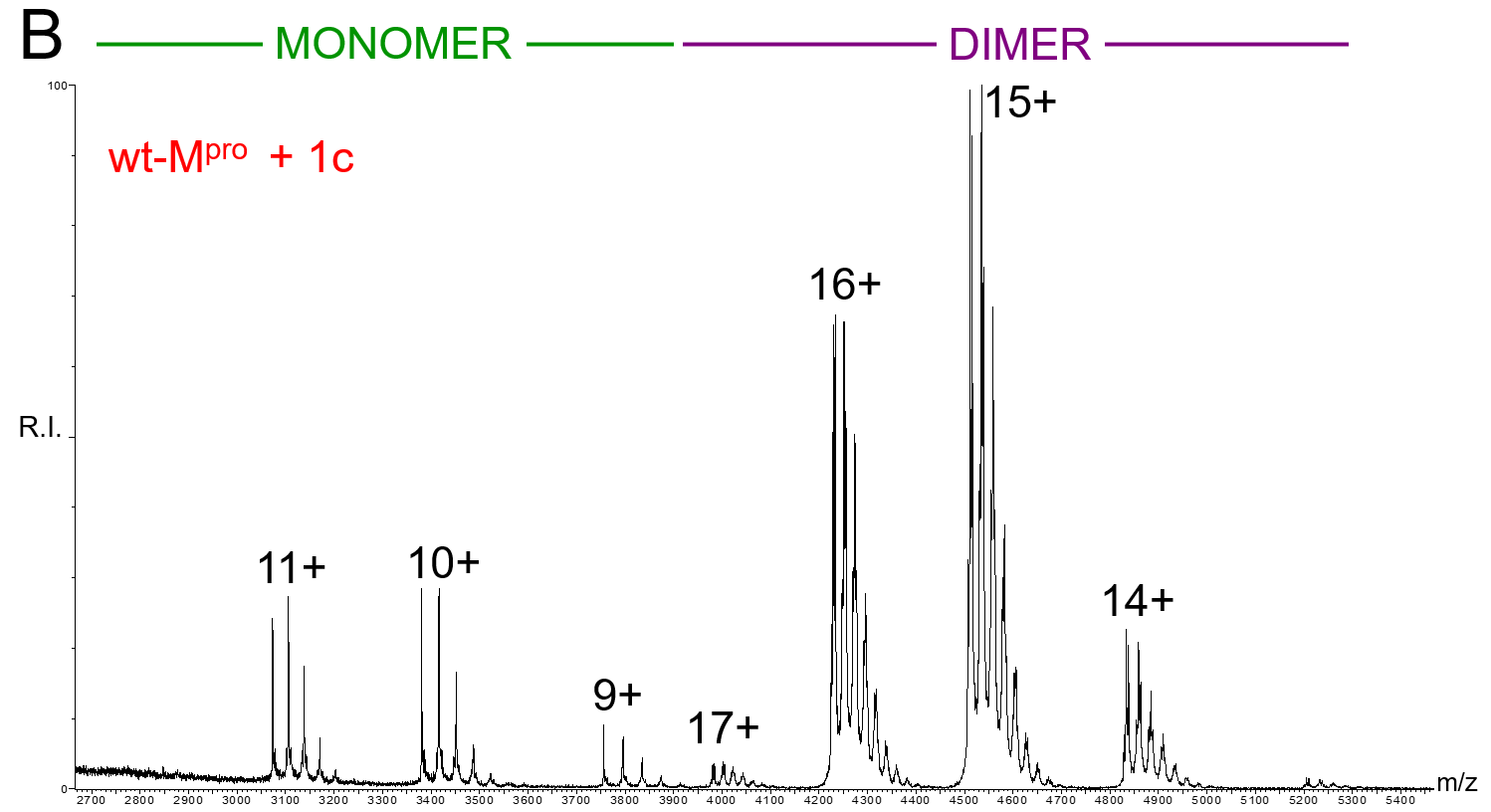 |
| 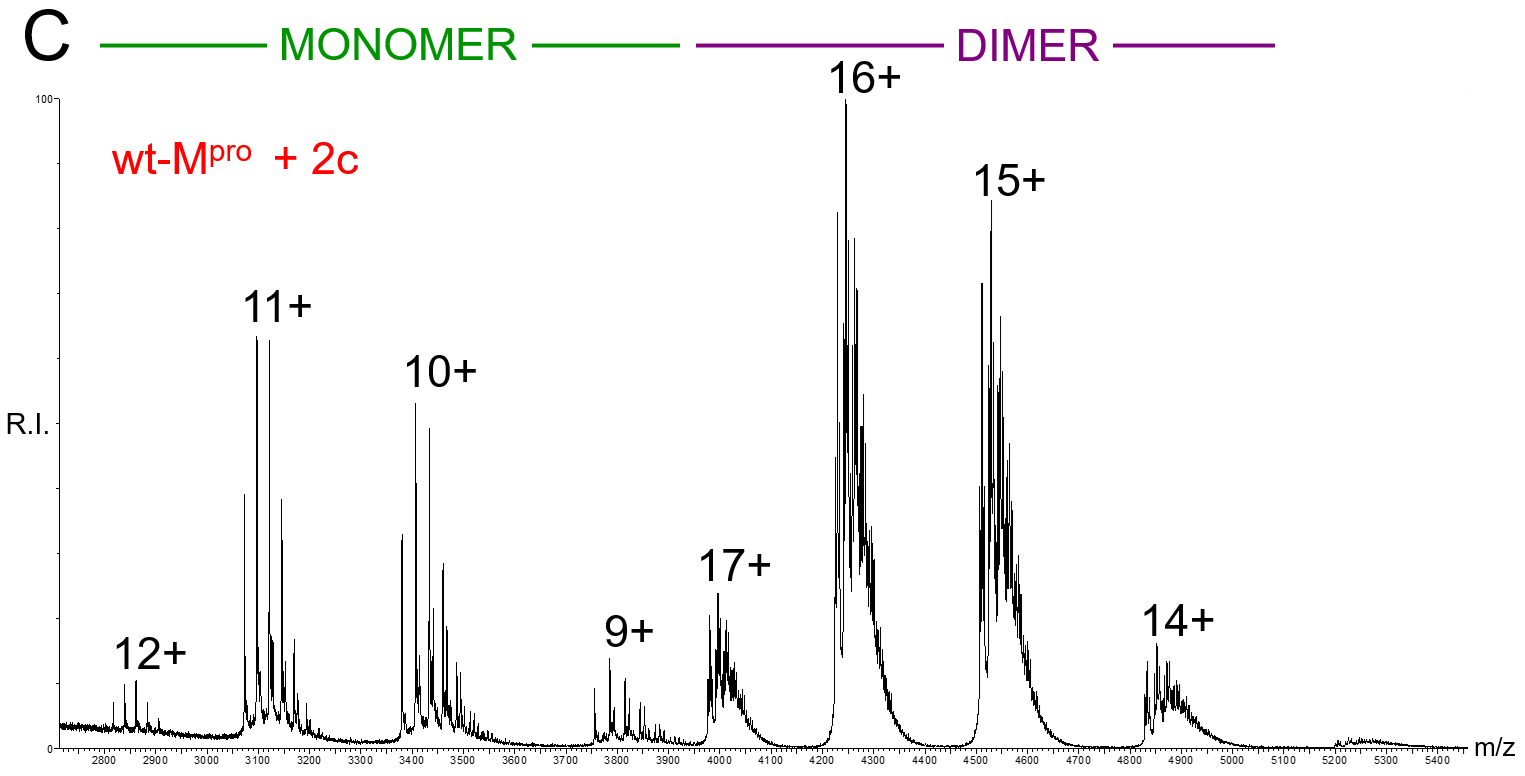 |
| 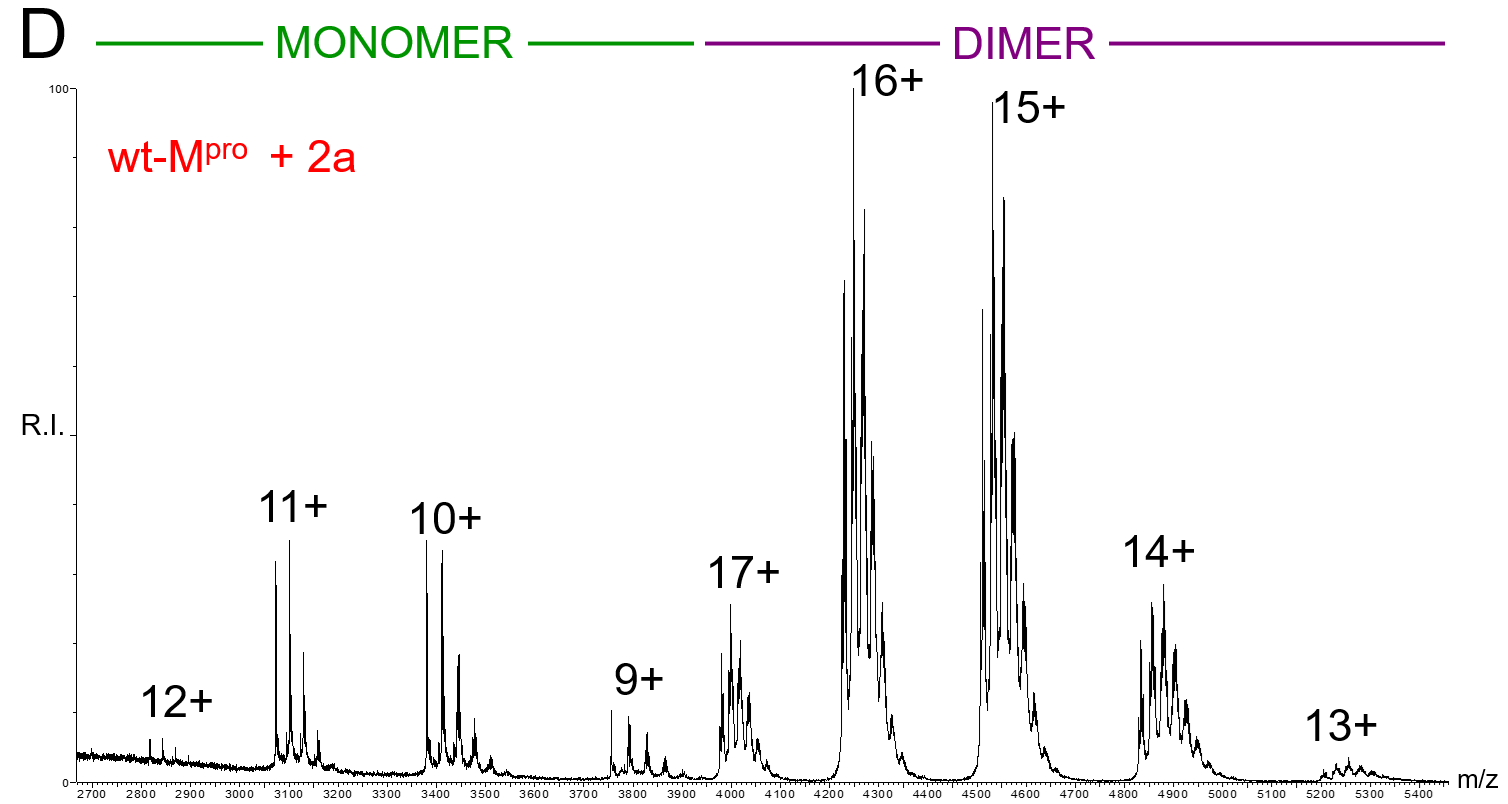 |
| 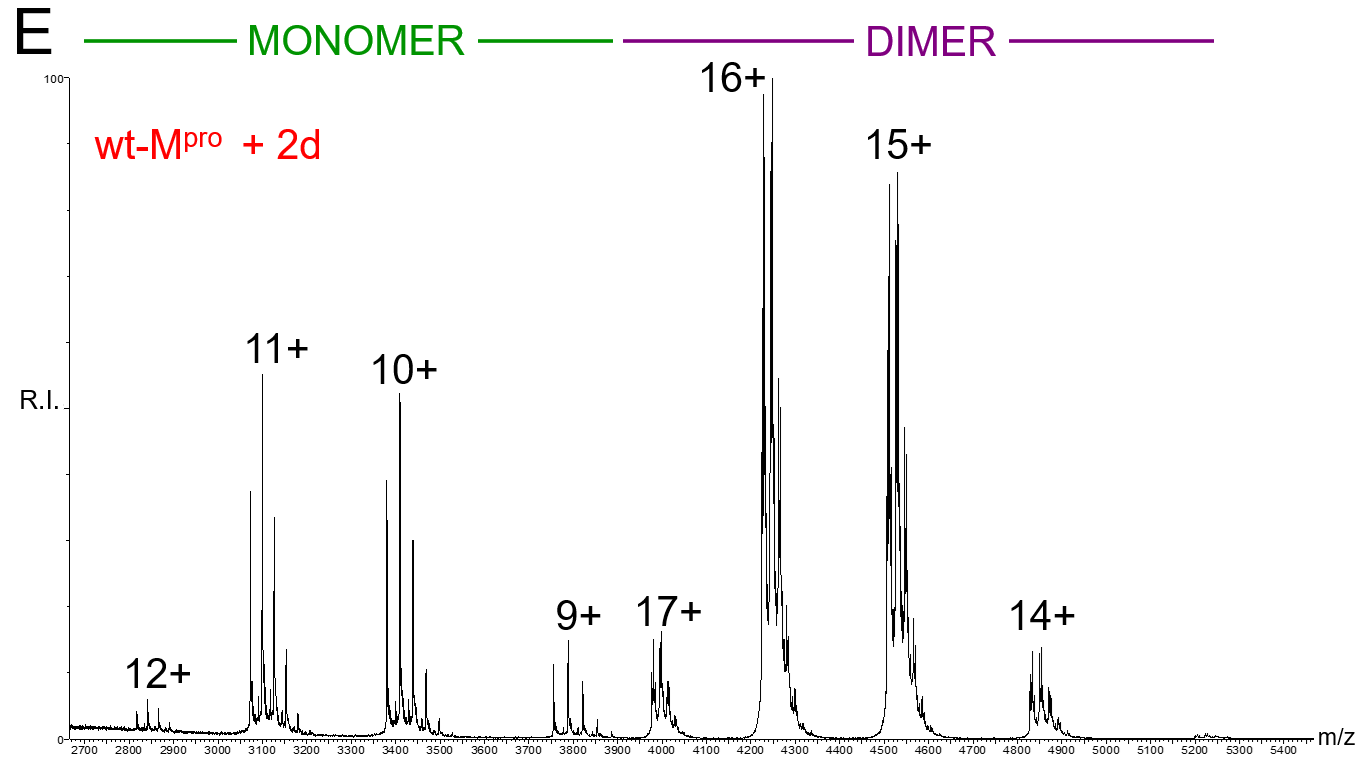 |
| 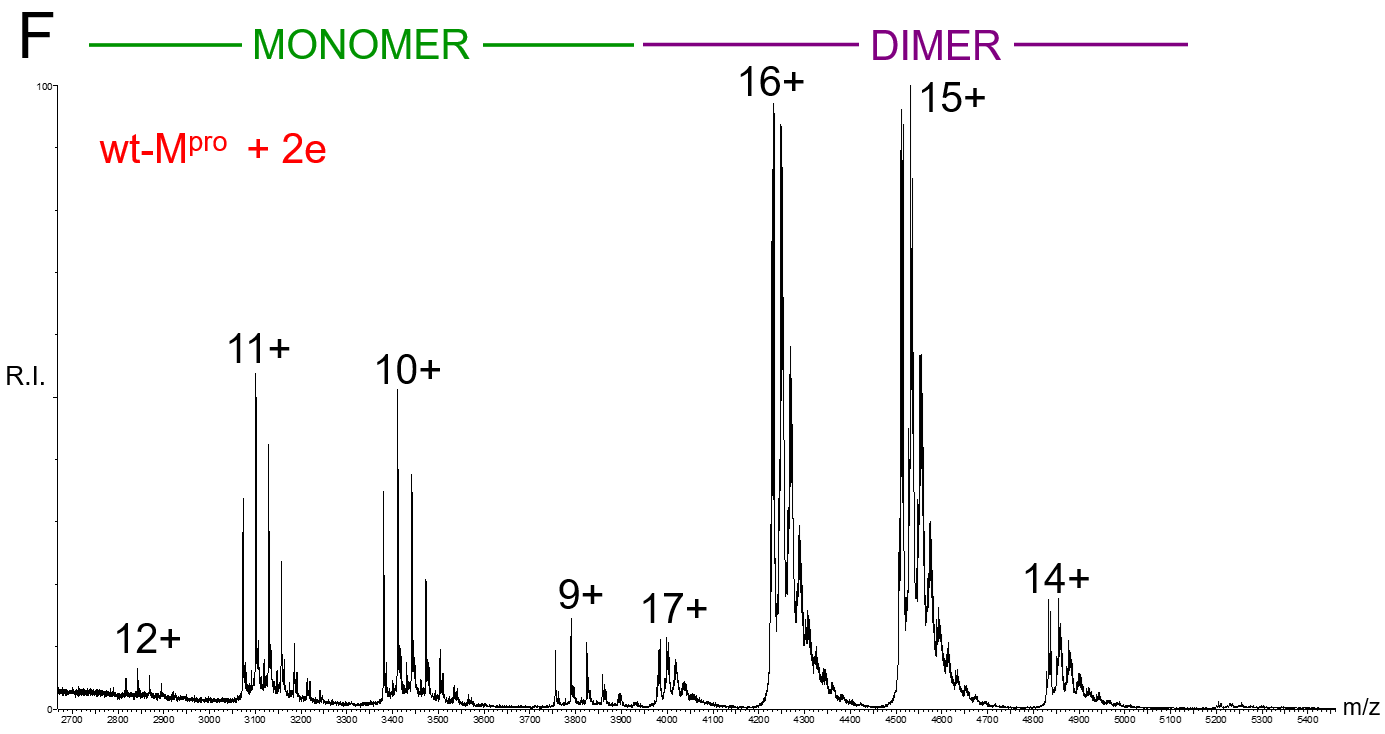 |
| 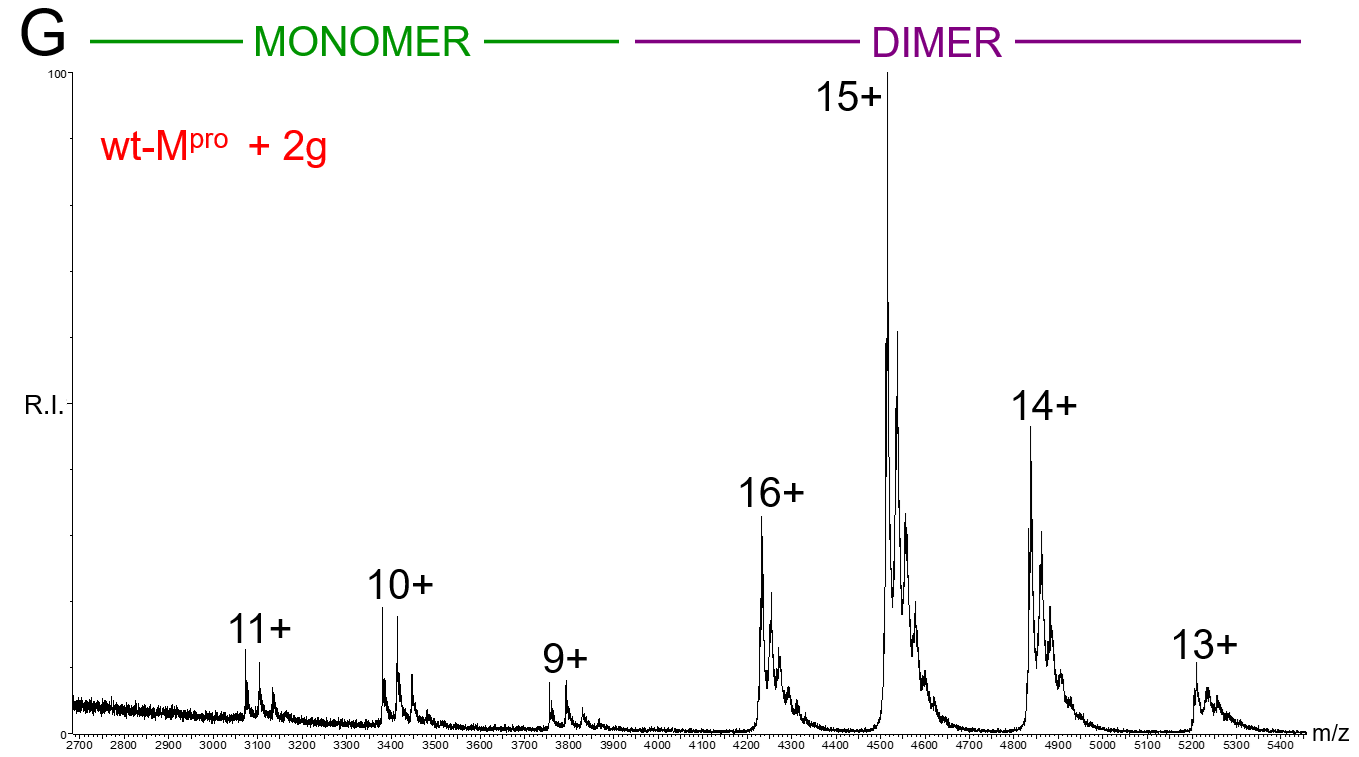 |
| 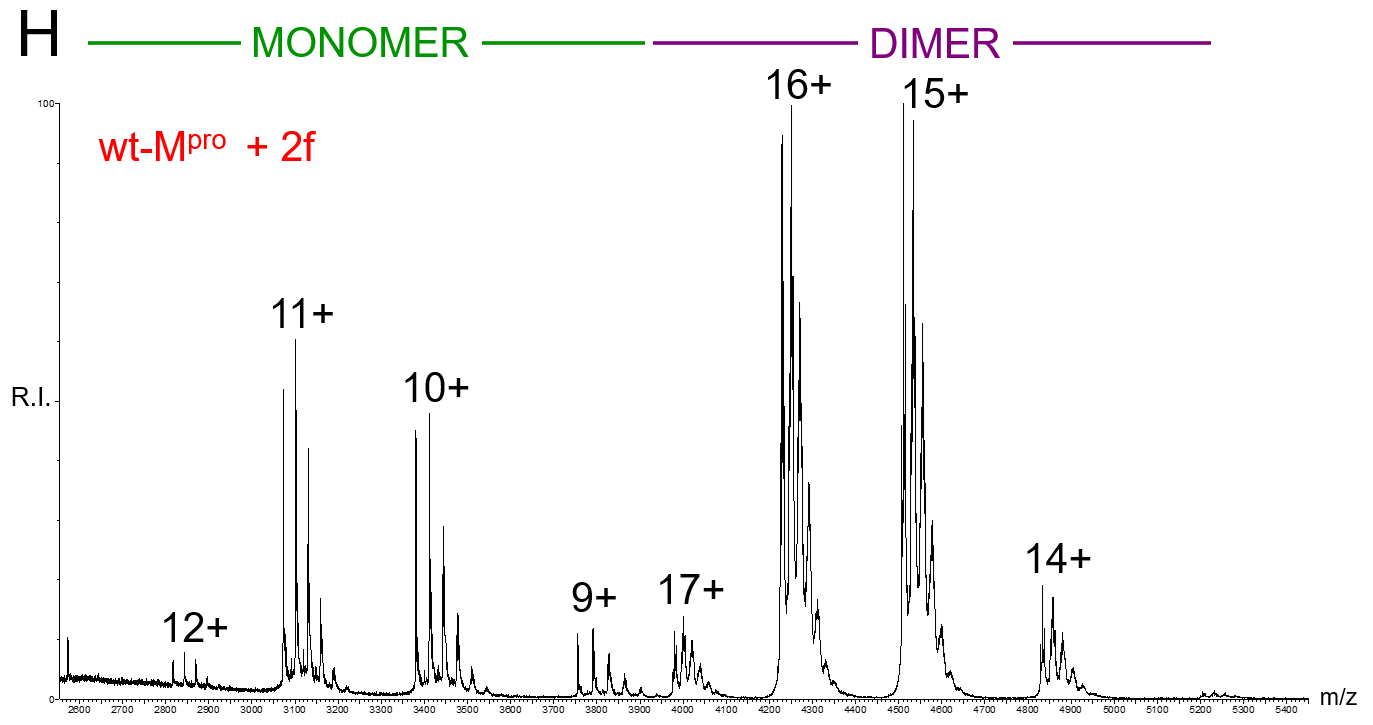 |

| **Figure S5. Representative native ESI-MS spectra of wt-M^pro^ in the presence of 5-fold molar excess of Ebselen or its derivatives.** The analysis was performed using 5 μM wt-M^pro^ (monomer concentration) in the presence of 25 μM of compound (compound-to-protein molar ration of 5:1). In each panel, the histogram (inset) shows the percentages of monomeric and dimeric conformations of wt-M^pro^, as calculated from the relative native mass spectrum. Panels (compound): **A**(**1a-Ebselen**), **B**(**1b**), **C**(**1c**), **D**(**2b**), **E**(**2c**), **F**(**2a**), **G**(**2d**), **H**(**2e**), **I**(**2g**) and **J**(**2f**).   \| 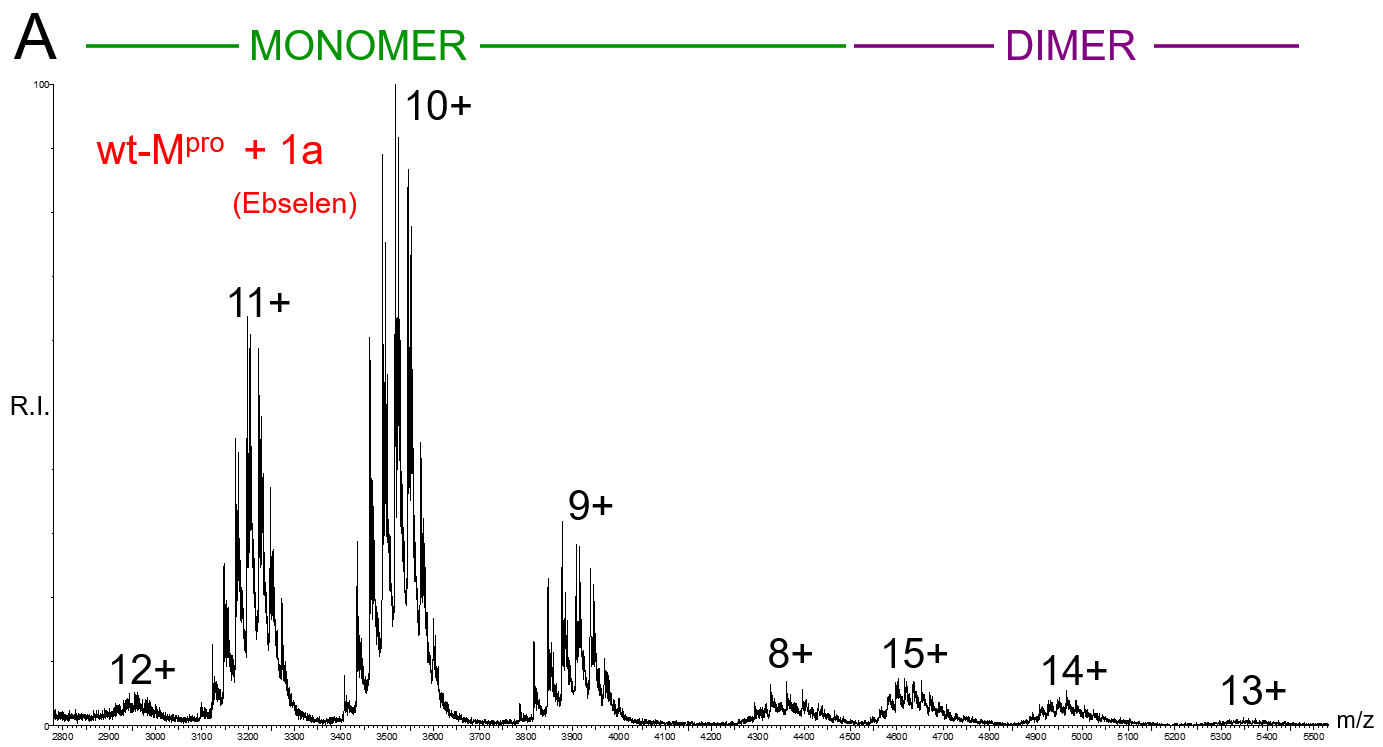 \| 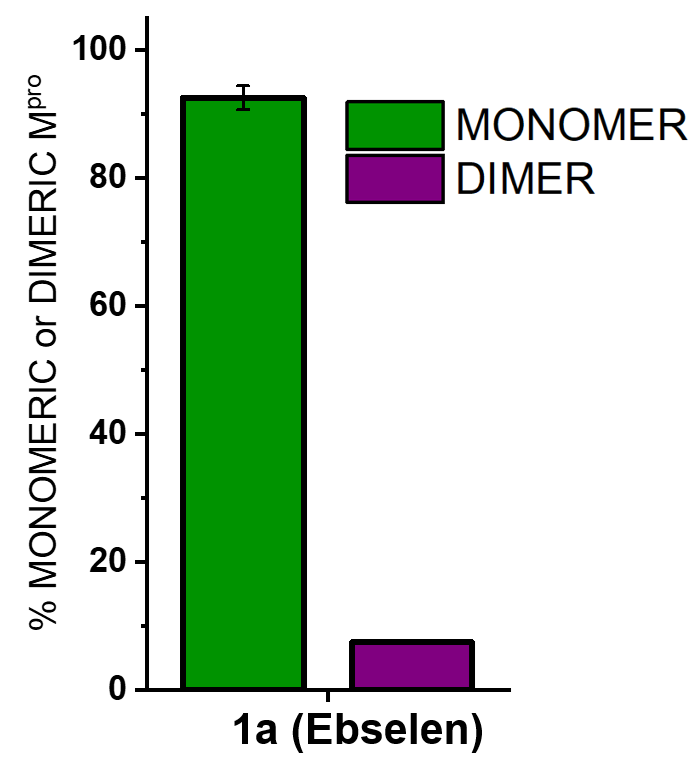 \| \| --- \| --- \| \| 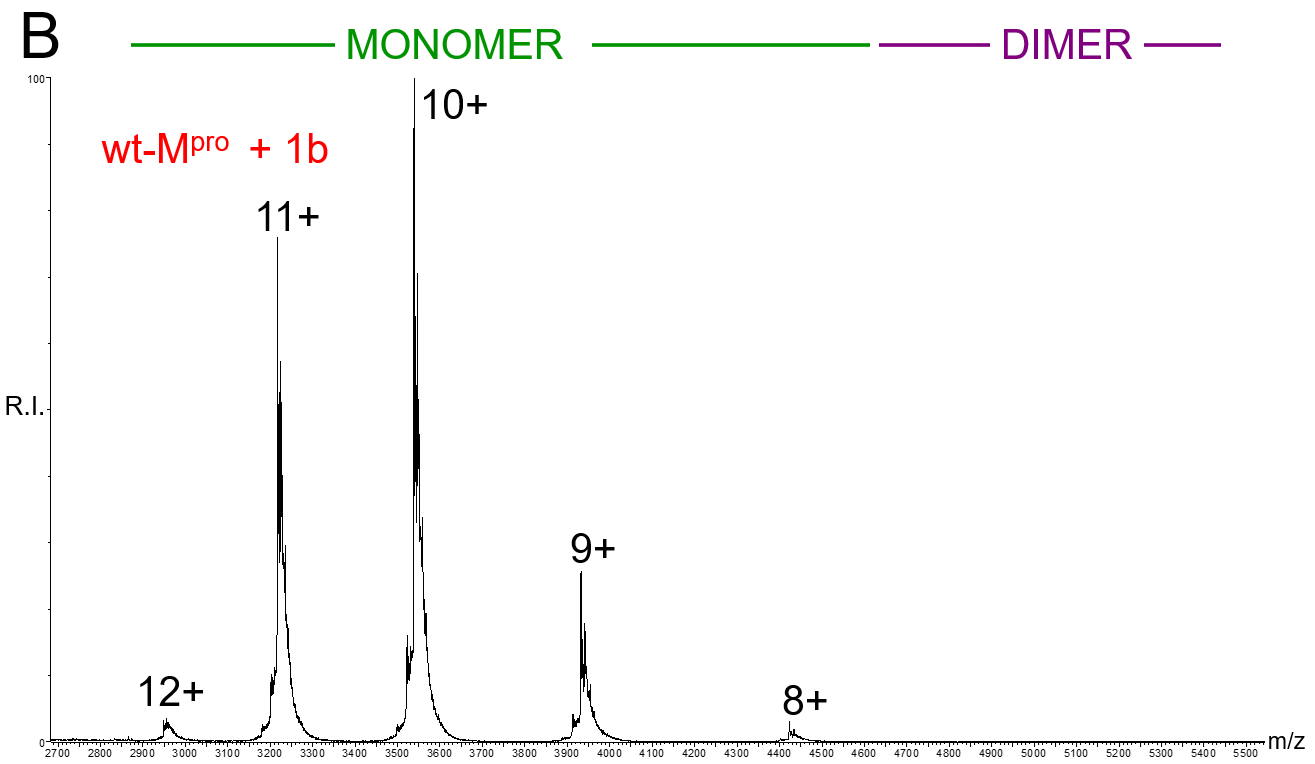 \| 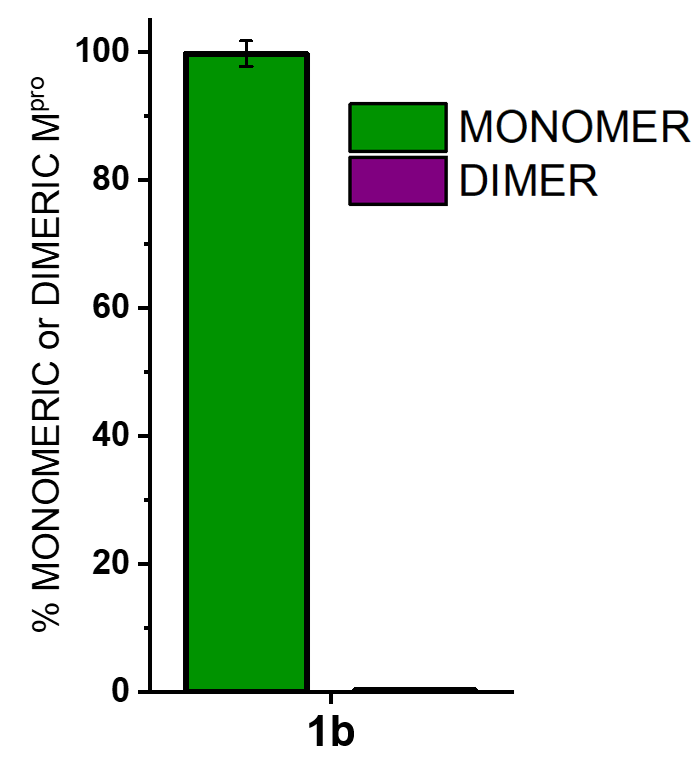 \| \| 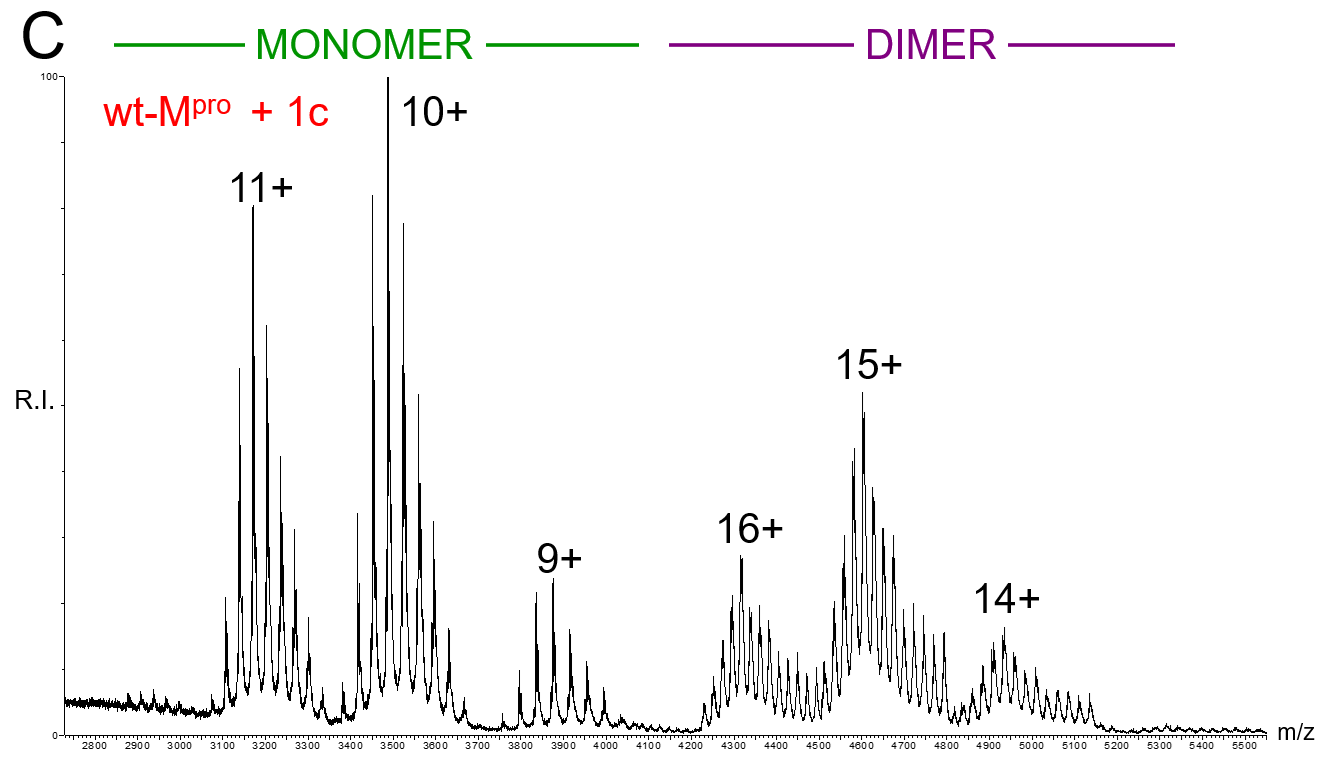 \| 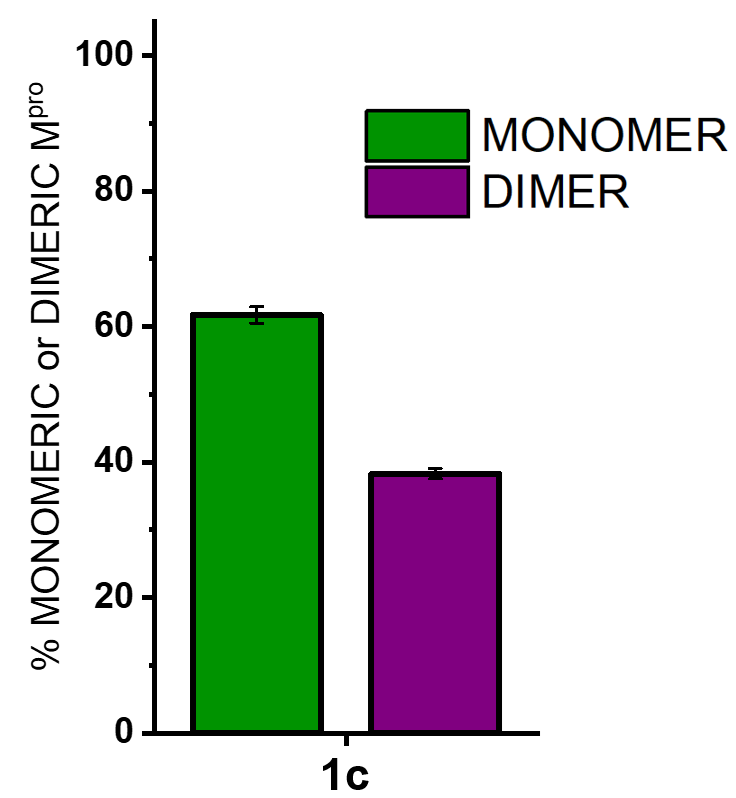 \| \| 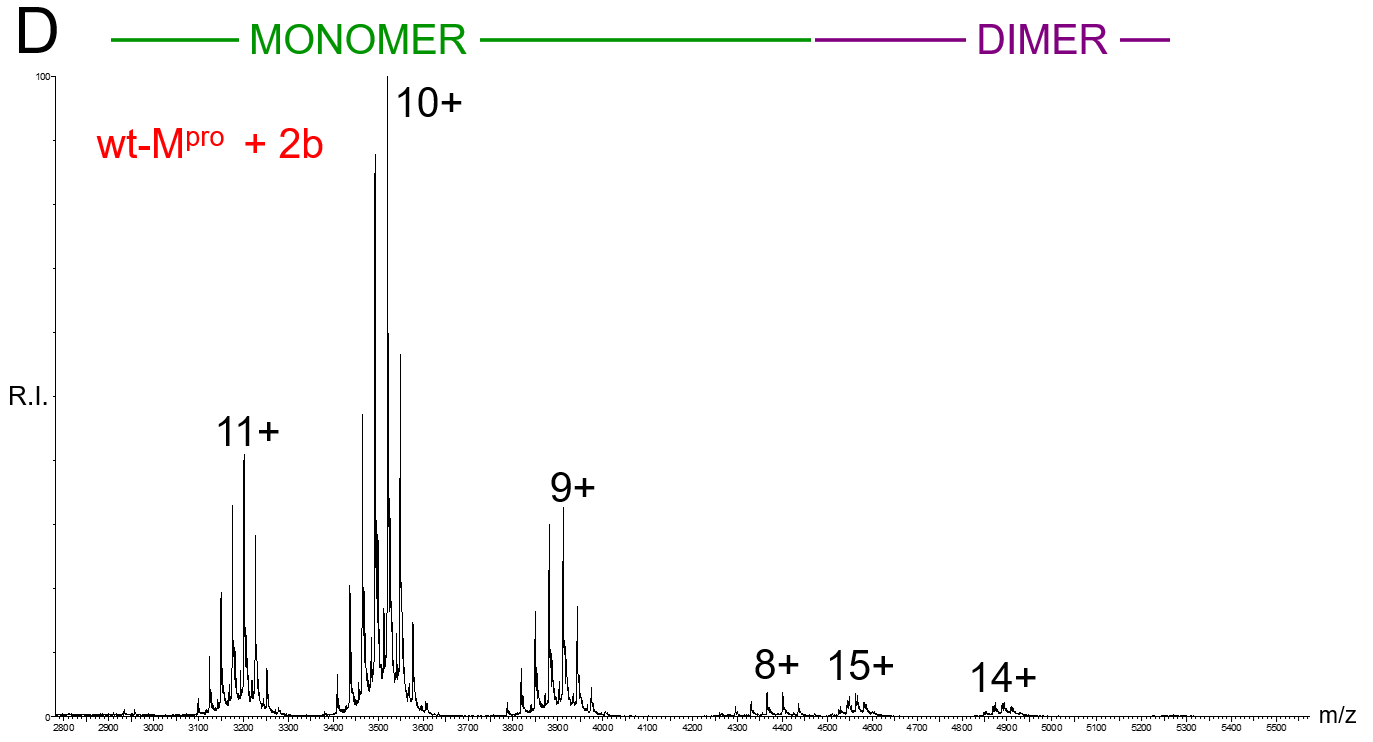 \| 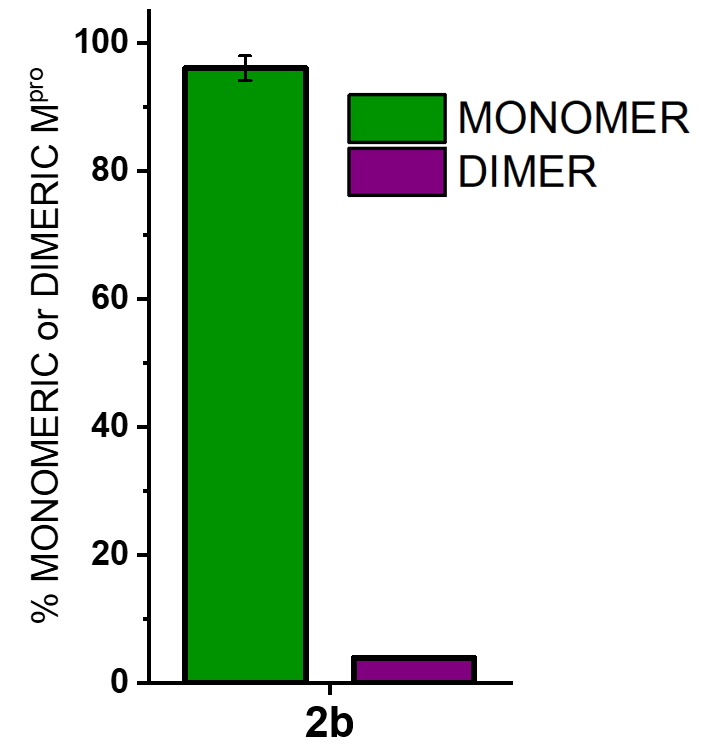 \| \| 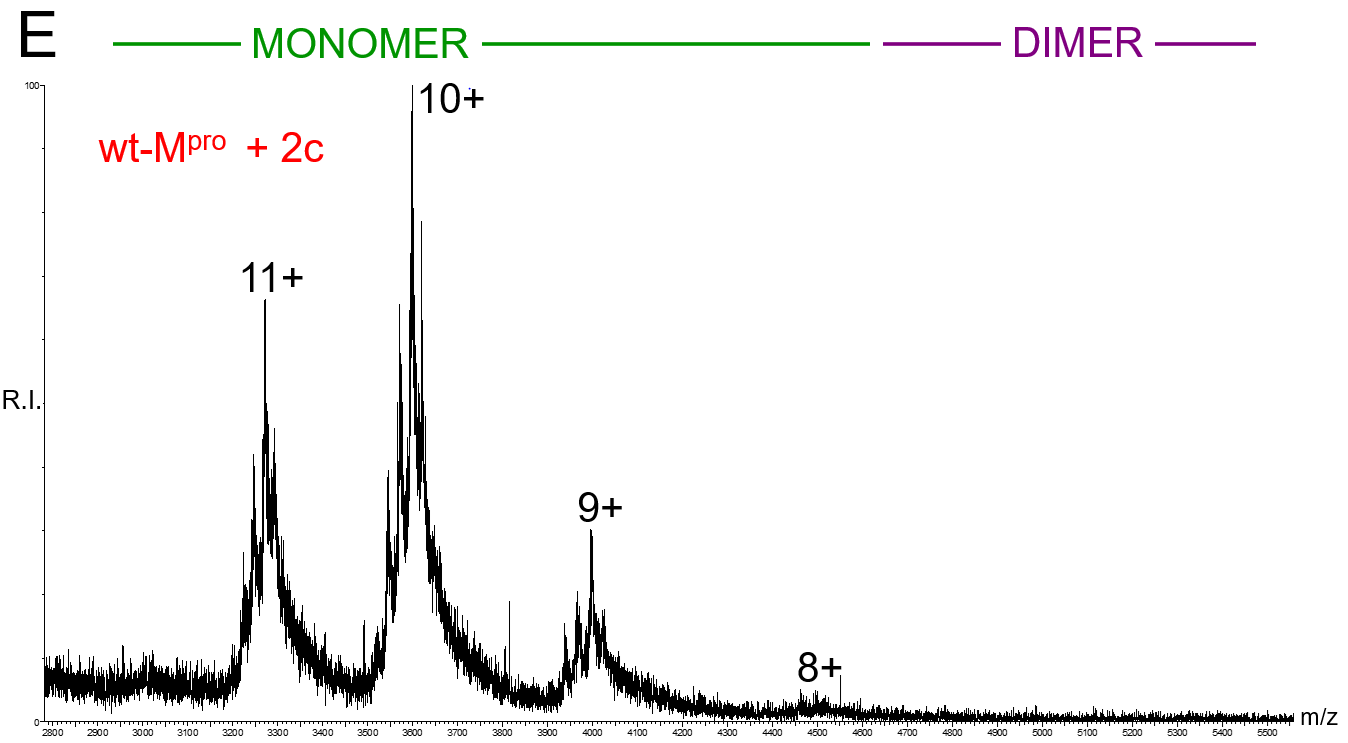 \| 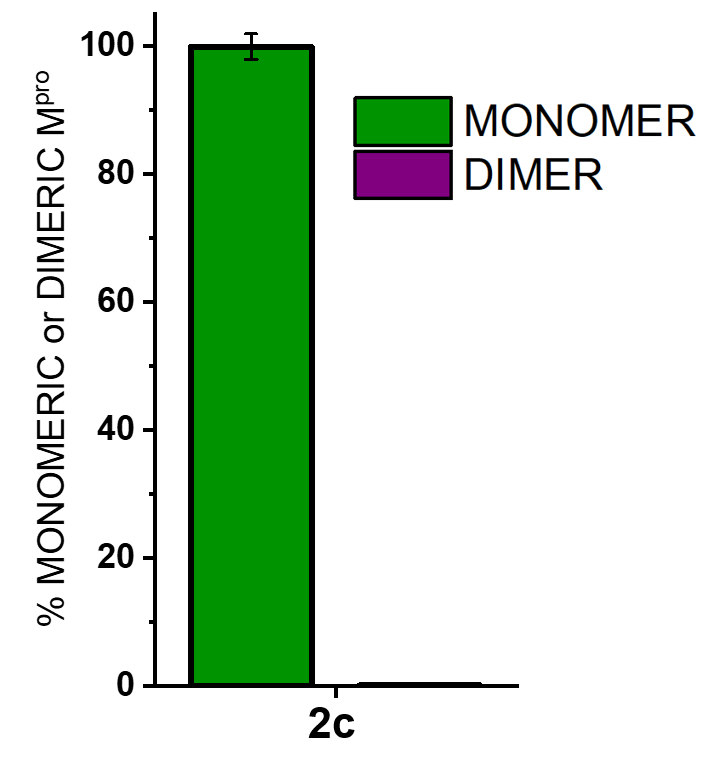 \| \| 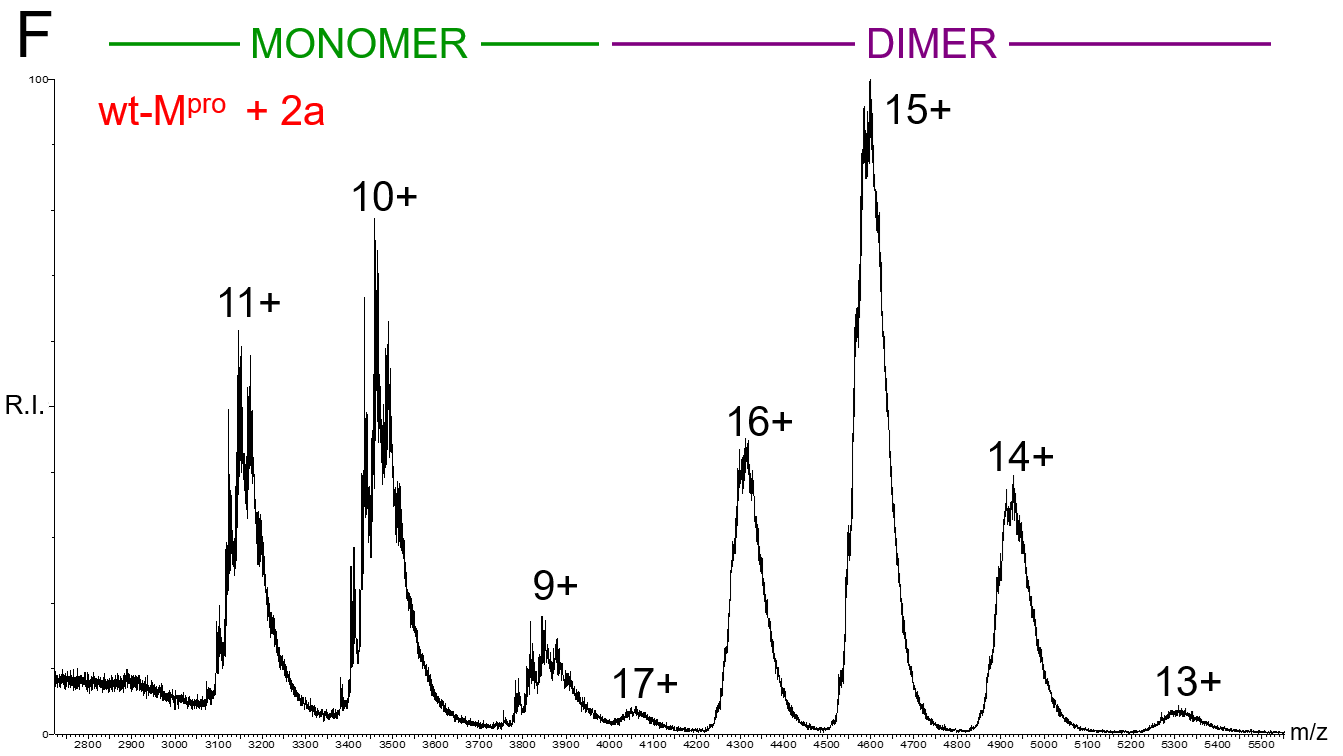 \| 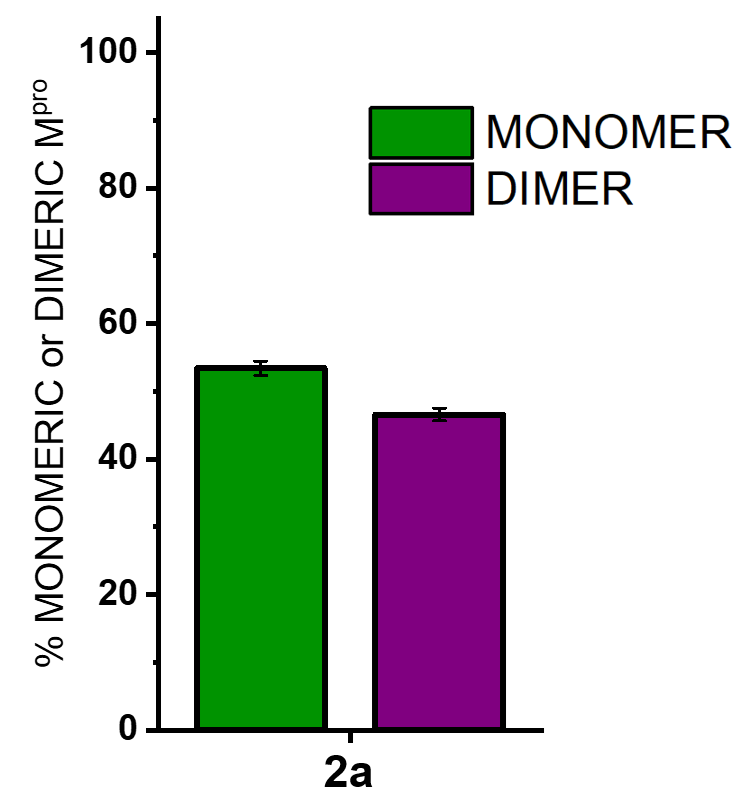 \| \| 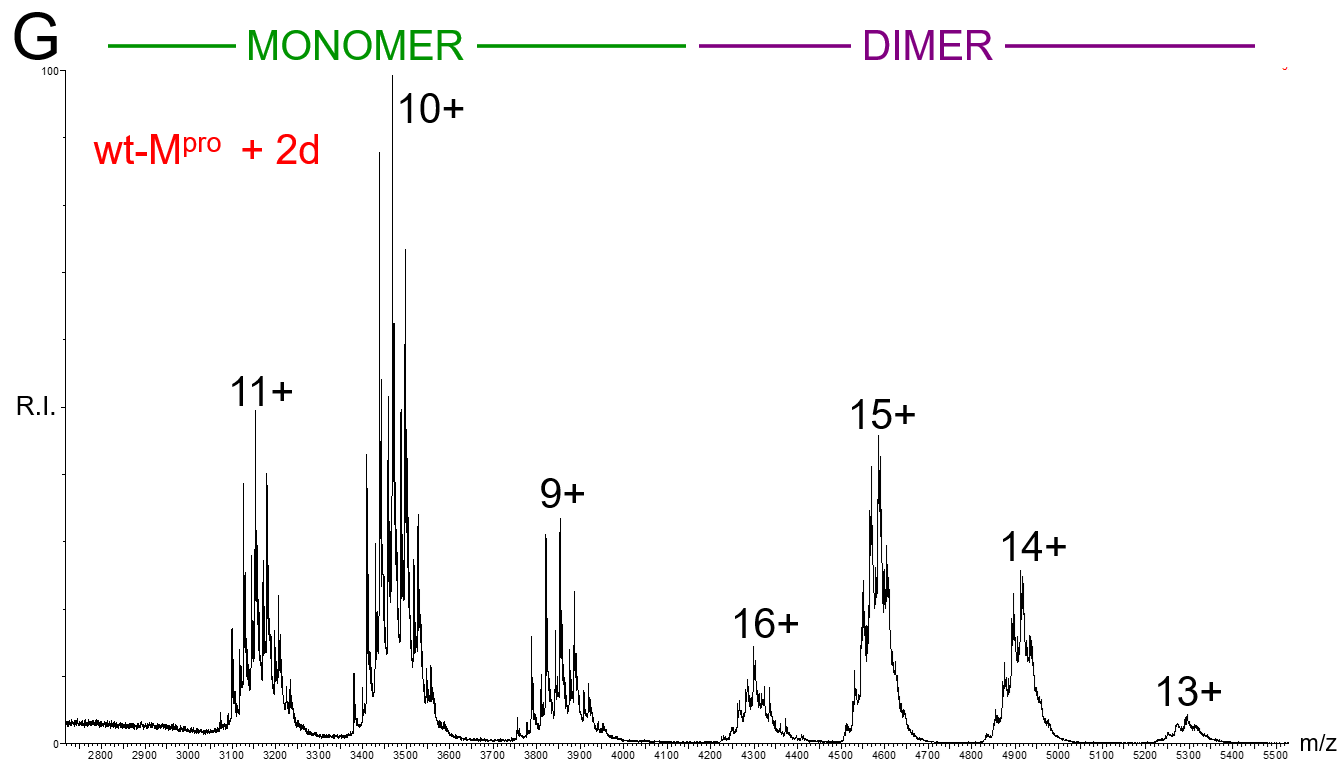 \| 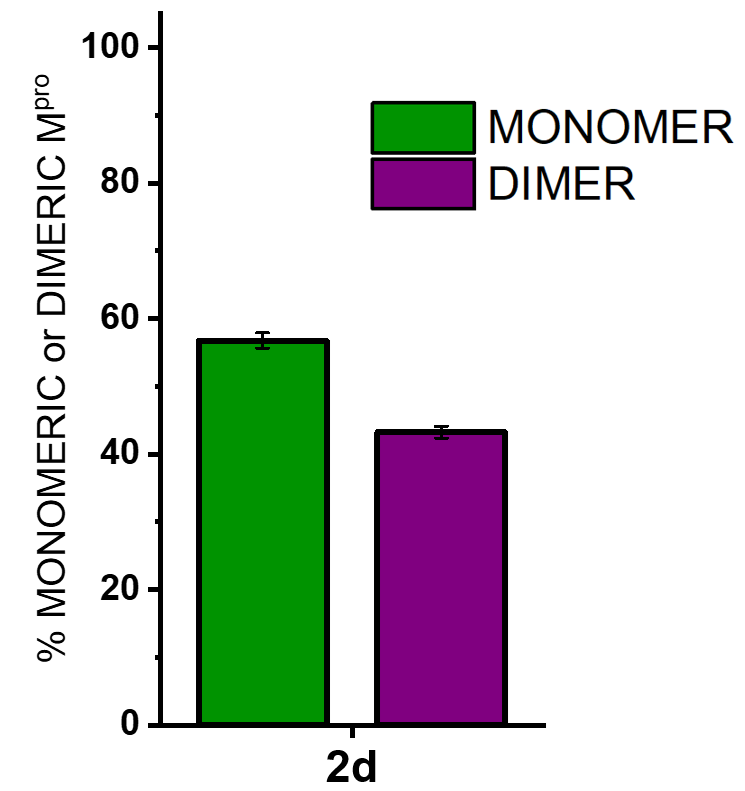 \| \| 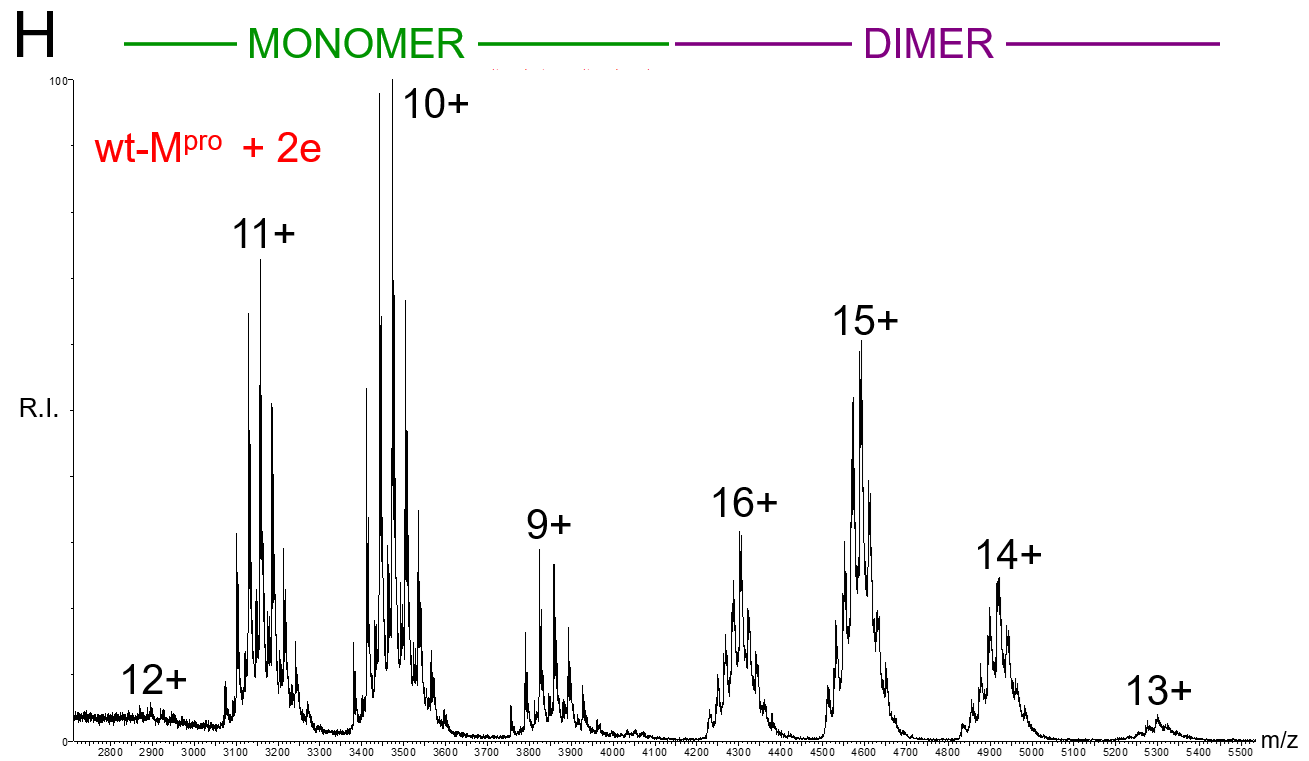 \| 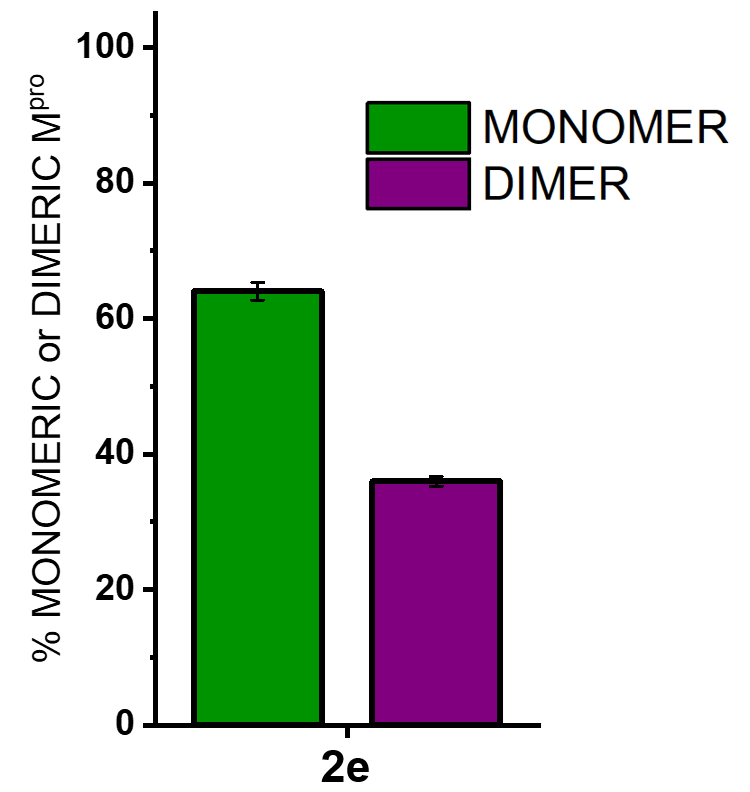 \| \| 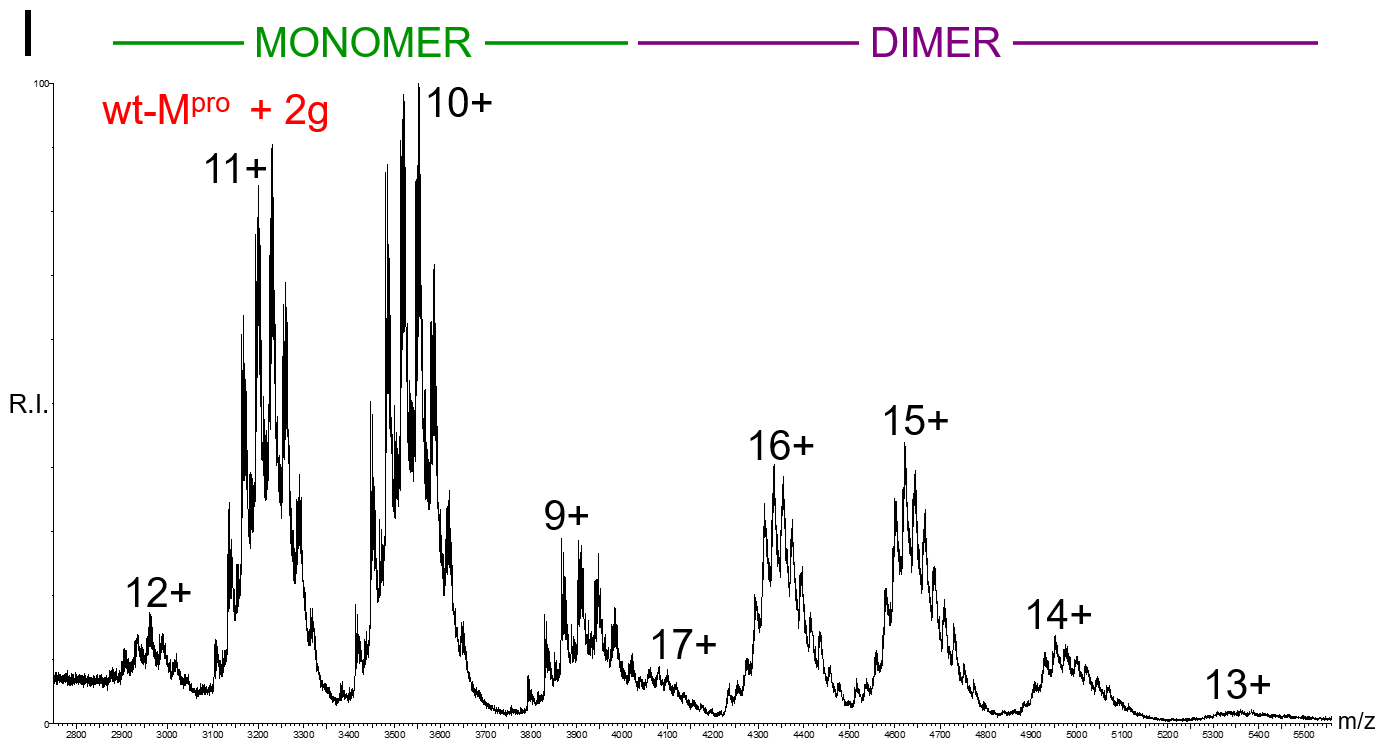 \| 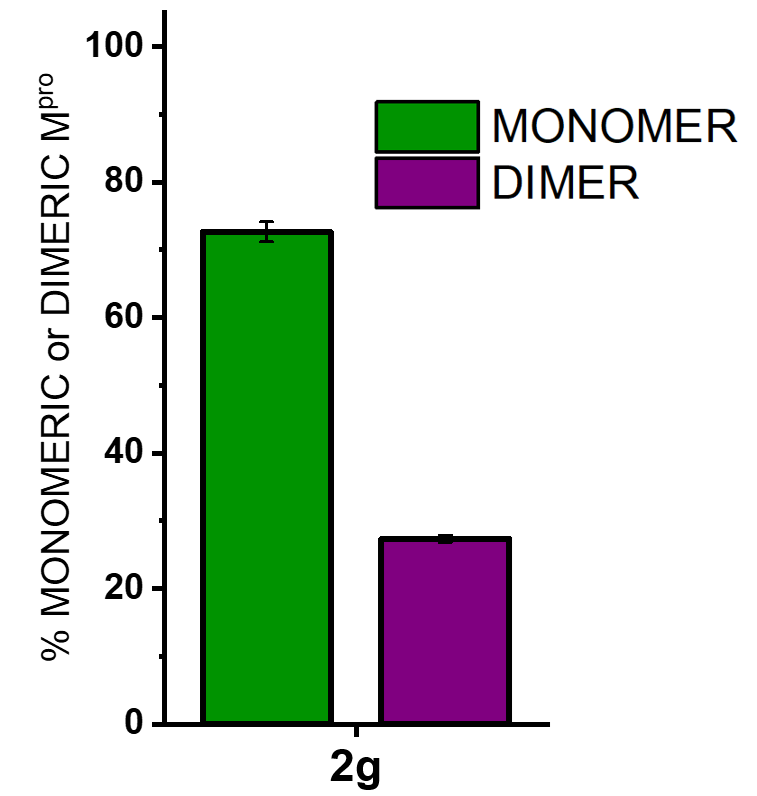 \| \| 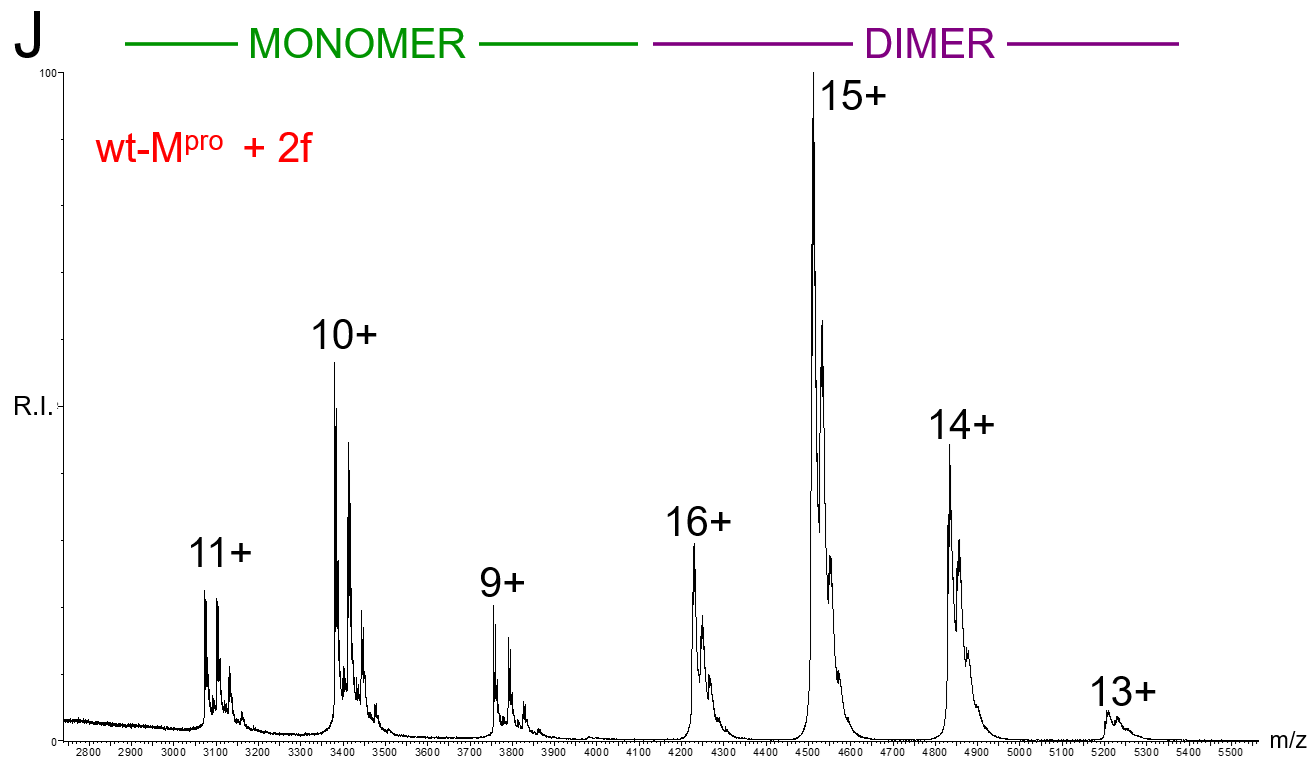 \| 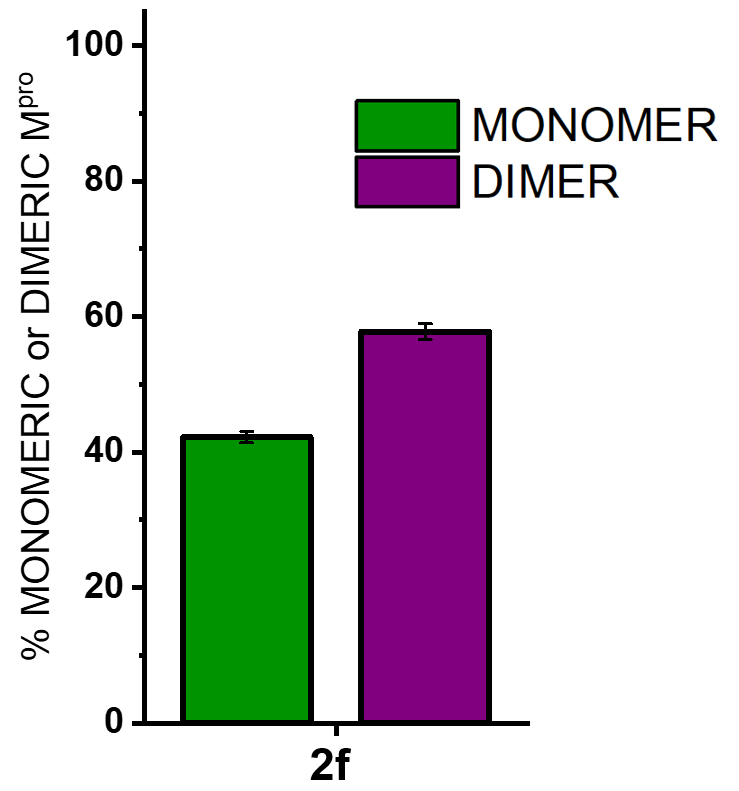 \| \|  \| \| |
| --- | --- | --- | --- | --- | --- | --- | --- | --- | --- | --- | --- | --- | --- | --- | --- | --- | --- | --- | --- | --- | --- | --- |

**Figure S6. Comparison of Solvent-Accessible Surface Area (SASA) of cysteine residues in each protomer of dimeric and monomeric SARS-CoV-2 wt-M^pro^.** Solvent-Accessible Surface Area (SASA, Å^2^) were calculated for the 12 cysteine (C) residues in a protomer of the free form of wt-M^pro^ (PDB 6Y2E) when part of the symmetric dimer (purple bars) or isolated (that is as a “monomer”, green bars) and their values are reported above each bar. To facilitate the association of the cysteine residues with their corresponding region in wt-M^pro^, the three structural domains of each protomer are indicated above the graph: domain I (residues 10-99), domain II (residues 100-182), and domain III (α-helical dimerization domain, residues 198-303).

| 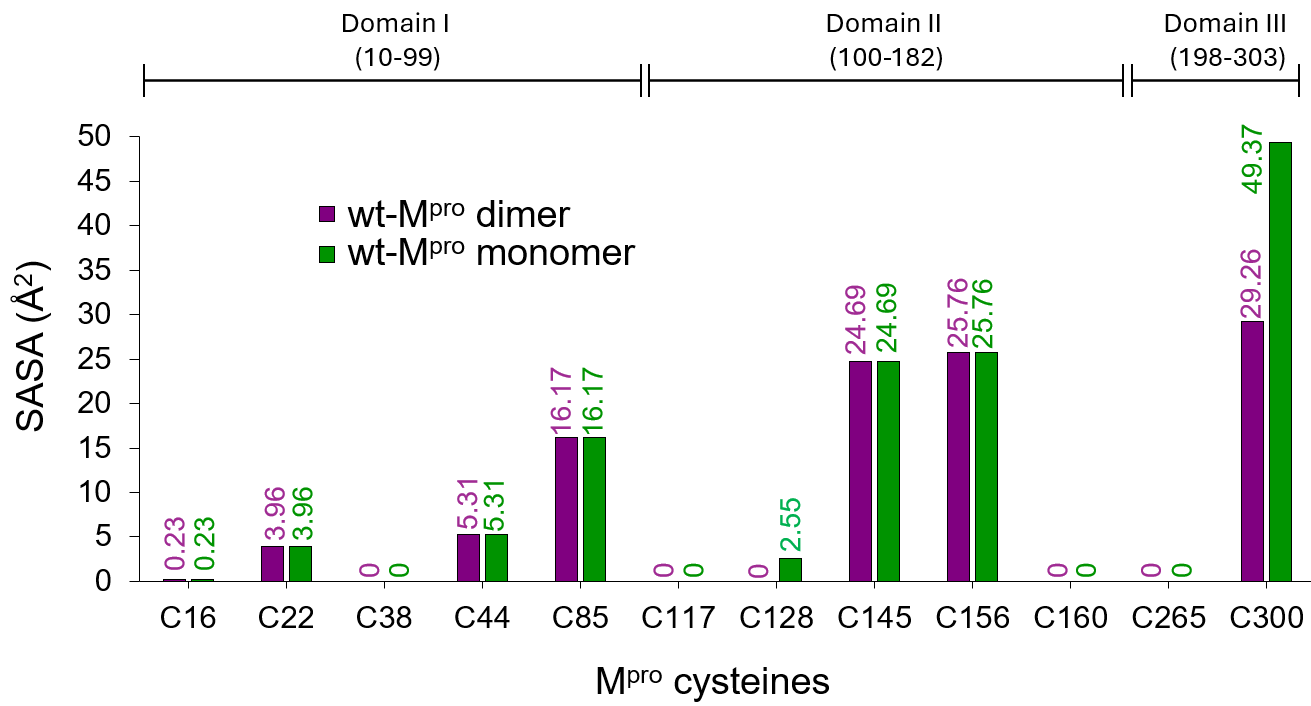 |
| --- |

**Figure S7. Antiviral activity of Ebselen and the N-acyl-derivatives 2b and 2c on Vero E6 cells infected with SARS-CoV-2.** The graph shows the percentage of viral infectivity upon incubation with Ebselen, **2b** and **2c**. Infection level for each concentration of the tested compounds was expressed as percentage in comparison to the untreated control (DMSO) cells. Concentrations of the compounds increases range from 0.39 μM to 50 μM. Data represent the mean ± SD of two independent experiments performed in duplicate.


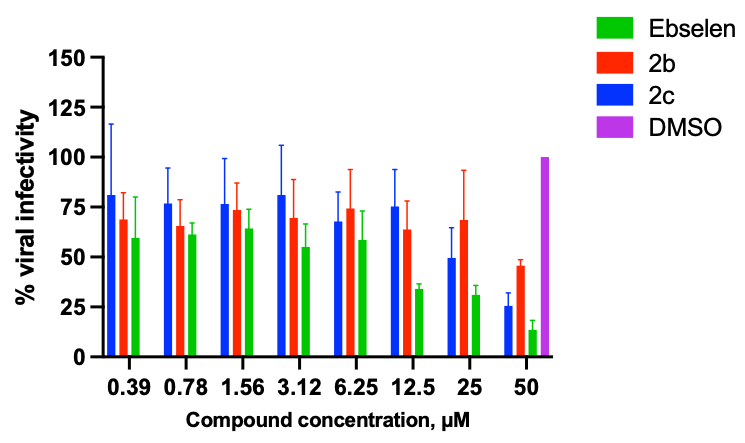

Supplement: Fabbian_et_al_SUPPLEMENTARY.docx [file IENZ_A_2604232_SM8747.docx]
